# Multi-Site Classification of Autism Spectrum Disorder Using Spatially Constrained ICA on Resting-State fMRI Networks

**DOI:** 10.3390/brainsci16020181

**Published:** 2026-01-31

**Authors:** Talha Imtiaz Baig, Junlin Jing, Peng Hu, Bochao Niu, Zhenzhen Yang, Bharat B. Biswal, Benjamin Klugah-Brown

**Affiliations:** 1 The Clinical Hospital of Chengdu Brain Science Institute, MOE Key Laboratory for Neuroinformation, School of Life Science and Technology, University of Electronic Science and Technology of China, No. 2006, Xiyuan Avenue, West Hi-Tech Zone, Chengdu 611731, China; talhaimbaig@gmail.com (T.I.B.); junlin.jing@med.uni-muenchen.de (J.J.); penghu01@outlook.com (P.H.); niubochao011716@163.com (B.N.); yangzhenzhen.yang@std.uestc.edu.cn (Z.Y.); bbiswal@gmail.com (B.B.B.); 2 Department of Radiology, Ludwig-Maximilians-University Munich, Marchioninistr. 15, 81377 Munich, Germany; 3 Department of Biomedical Engineering, New Jersey Institute of Technology, 619 Fenster Hall, Newark, NJ 07102, USA

**Keywords:** Autism Spectrum Disorder (ASD), multi-site neuroimaging, brain connectivity, network analysis, spatial constraint ICA, machine learning, classification, variability reduction, feature extraction, support vector machine

## Abstract

**Background/Objectives**: Autism Spectrum Disorder (ASD) is a complex neurodevelopmental condition characterized by differences in social communications and restricted, repetitive patterns of behaviors and interests, affecting approximately 1% of children globally. While functional magnetic resonance imaging (fMRI) has provided insights into altered brain connectivity patterns in ASD, classification based on neuroimaging remains a challenging due to the heterogeneity of the disorder and variability in imaging data across sites. This study employs a network-based approach using large-scale, multi-site rs-fMRI dataset from the Autism Brain Imaging Data Exchange (ABIDE I and II) to classify ASD and healthy controls using machine learning. **Methods**: A semi-blind Independent Component Analysis method, specifically the spatial constraint reference ICA, is applied to identify functional brain networks, and the ComBat harmonization technique is used to address site-specific variability across 11 independent datasets, ensuring consistency in feature representation. Support Vector Machines (SVMs) are employed for classification, focusing on three key networks: the Default Mode Network (DMN), Sensorimotor Network (SMN), and Visual Sensory Network (VSN). **Results**: The results demonstrate high classification accuracy, with the VSN achieving the highest performance (83.23% accuracy, 87.90% AUC), followed by the DMN (81.43% accuracy, 84.53% AUC) and the SMN (80.52% accuracy, 84.96% AUC), positioned with their recognized roles in social cognition and sensory–motor processing, respectively. **Conclusions**: The integration of ICA-based feature extraction with ComBat harmonization significantly improved classification accuracy compared to previous studies. These findings point out the potential of network-based approaches in ASD classification and point out the importance of integrating multi-site neuroimaging data for identifying reproduceable network-level features.

## 1. Introduction

Autism Spectrum Disorder (ASD) is a persistent neurodevelopmental disability characterized by social interaction difficulties and repetitive behaviors [1]. The significant variability in neurocognitive features across autism from childhood to adulthood point out the complexity of identifying consistent neurobiological markers [2]. According to the World Health Organization (WHO), approximately 1 in 100 children globally is affected by ASD [3]. Recently, ASD diagnosis has relied on behavioral assessments [4,5,6] which are accurate but time-consuming and resource-intensive, and must be carried out by an expert.

Neuroimaging techniques offer promising paths for identifying effective physiological features associated with ASD by examining brain connectivity patterns. Functional magnetic resonance imaging (fMRI) is widely known for its ability to investigate these dynamics [7,8]. fMRI studies have shed light on the neurological basis of ASD, revealing altered functional connectivity in key brain regions such as the cerebellum, amygdala, and prefrontal cortex [9,10]. Given the complexity and heterogeneity of ASD, data-driven methods like independent component analysis (ICA) [11,12] enable brain signals to be decomposed into distinct, independent sources, allowing researchers to identify functional networks. This approach is particularly valuable in ASD research, providing a more comprehensive understanding of the disorder’s neural mechanisms.

Machine learning in healthcare has also emerged as a powerful data-driven tool in neuroimaging research [13,14]. Supervised methods including Support Vector Machines (SVMs) are frequently employed as diagnostic tools [15]. SVMs are particularly effective due to their robustness against overfitting and their ability to handle high-dimensional data efficiently [16,17]. In ASD classification, when processing data that is nonlinear and is also nonlinear in the original feature space, SVM can employ the kernel function to project the dataset into a larger space, where it becomes discrete [18,19].

First, although large multi-site repositories such as ABIDE provide increased statistical power, many existing studies have either focused on single-site analyses [20,21] or pooled multi-site data without explicit harmonization procedures [22]. This practice has been shown to reduce model generalizability due to scanner- and site-related variability [23,24,25,26]. Second, a large proportion of ICA-based ASD studies rely on unconstrained ICA approaches or fully data-driven component estimation, which can produce subject-specific or dataset-specific components. Such variability undermines the reproducibility and cross-site comparability of functional networks, particularly in large heterogeneous datasets [27]. Third, although modern machine learning and deep learning models have achieved moderate classification performance using ABIDE data [22], many of these approaches provide limited insight into network-level contributions, restricting their interpretability in the context of large-scale functional brain organization relevant to ASD.

To address these limitations, the present study integrates three complementary methodological components: (i) spatially constrained ICA using validated network templates to ensure consistent component extraction across subjects and sites; (ii) ComBat harmonization to reduce site-specific variability while preserving biologically meaningful signals; and (iii) a network-specific machine learning framework evaluated across 11 independent sites. The current investigation focuses on the default mode, sensorimotor, and visual networks, which have been repeatedly implicated in ASD-related alterations in social cognition, sensory processing, and motor function [28,29,30].

The primary objective of this research is to develop a reproducible, interpretable, and multi-site harmonization-driven analytical framework that advances beyond conventional ICA- or SVM-based approaches to Autism Spectrum Disorder (ASD) classification [31]. Specifically, this study aims to (1) mitigate multi-site variability through the application of a robust data harmonization strategy; (2) evaluate the discriminative contribution of major large-scale functional brain networks to ASD classification; and (3) assess the reliability and generalizability of machine learning-based classification performance across independent sites. All technical aspects, including functional network selection, ICA methodology, and harmonization procedures, are detailed in the Section 2.

## 2. Materials and Methods

Five key steps were incorporated to develop a methodology based on network analysis: (1) acquisition of data; (2) preprocessing; (3) spatial constraint ICA analysis; (4) harmonization using ComBat; (5) classifying ASD and HC into two categories.

### 2.1. Data Acquisition

Our study utilized publicly available datasets from the Autism Brain Imaging Data Exchange (ABIDE-I and ABIDE-II) repository (http://fcon_1000.projects.nitrc.org/indi/abide/ (accessed on 15 August 2025)) [32,33]. This dataset includes contributions from 38 different sites worldwide, encompassing a total of 2264 participants, with 1083 subjects diagnosed with ASD and 1181 healthy controls. Each contributing site confirmed ASD diagnoses using standardized clinical assessments [16].

To enhance the dataset quality, the study implemented data cleaning and preprocessing steps. Initially, sites with limited data on ASD subjects, such as CMU from ABIDE-I, were excluded. Data from individuals with excessive head motion (greater than 2 mm) or rotations exceeding 2 degrees were also removed [34]. Data from two ASD-only sites (KUL and NYU2 from ABIDE-II) were excluded. Approximately 300 participants were removed due to head motion, noise, or missing features data. Subjects with fewer time points were also excluded.

Following standard preprocessing procedures, data from 35 sites, comprising 1750 participants, were initially retained. To ensure reliable ICA decomposition and effective site-specific harmonization, only sites with at least 50 participants (including both individuals with ASD and healthy controls) were included in the final analysis. Prior research [35] has demonstrated that small site-level sample sizes in multi-site studies can lead to unstable ICA component estimation, inflated variance, and compromised model generalizability. Adequate per-site sample sizes are also critical for high-dimensional machine learning approaches, as they help mitigate overfitting and support robust within-site cross-validation. Applying this criterion yielded a final dataset of 996 participants from 11 sites, including 451 individuals with ASD and 545 typically developing controls. Participant ages ranged from 5 to 64 years, and the sample exhibited a male predominance, consistent with the sex distribution commonly observed in ASD research. This curated dataset was used to validate and quantitatively evaluate the performance of the proposed method.

### 2.2. fMRI Data Preprocessing

Resting-state fMRI data were preprocessed using the DPARSFA toolbox (version V7.0; http://www.rfmri.org; State Key Laboratory of Cognitive Neuroscience and Learning, Beijing Normal University, Beijing, China) [36,37] in MATLAB (version R2022b; The MathWorks Inc., Natick, MA, USA). The preprocessing was performed according to the following procedure: (1) removing the initial 10 volumes to avoid the impact of uncertainty of system scanning; (2) performing slice time correction; (3) performing three-dimensional realignment for head movement correction; (4) removing non-brain tissues using BET; (5) performing T1 weighted co-registration; (6) using DARTEL for segmentation; (7) global signal regression was skipped because of apprehension about negative correlations [38,39,40]; (8) data was normalized with [3 × 3 × 3] mm voxel size to MNI space [41]; (9) spatial smoothing was performed. So, 996 subjects from 11 different sites including 451 ASD patients and 545 HC controls were preprocessed.

### 2.3. Spatial Constraint ICA

This study employed spatially constrained ICA using predefined templates [11,42], which substantially reduced sensitivity to initialization and model-order variability compared to unconstrained ICA. The analysis was performed independently across 11 sites and on the harmonized dataset, yielding consistent extraction of the same 28 components. While multiple random initializations were not explicitly tested, prior work has demonstrated the improved stability of this approach.

Following preprocessing, a group-level average brain mask was generated across all sites to ensure a consistent feature space for subsequent analyses. Semi-blind spatially constrained independent component analysis (sICA) [43] was then performed using the GIFT toolbox (Group ICA of fMRI Toolbox, version 4.0.4.11; www.nitrc.org/projects/gift; The Mind Research Network, Albuquerque, NM, USA) (accessed on 30 August 2025)). Prior methodological work indicates that a minimum of approximately 20 components is required to obtain meaningful intrinsic connectivity networks, whereas model orders exceeding 100 components can substantially reduce ICA reproducibility [35,44]. Based on these considerations, the present study extracted 28 predefined intrinsic connectivity network (ICN) spatial maps.

Spatial constraints were derived from well-established resting-state network templates provided by the NeuroMark atlas within the GIFT framework. These templates were originally estimated from large, independent healthy control datasets and have been validated to represent stable and reproducible functional brain networks. Incorporating these spatial priors into the ICA framework ensures consistent extraction of homologous components across subjects and sites, thereby improving cross-site comparability and reproducibility.

For each ICN, normalized spatial maps were obtained by applying voxel-wise z-score normalization. Within each component mask, mean voxel intensity values were computed to generate a numerical feature vector for each component per subject. Prior to network-level filtering, this procedure yielded 28 component-level feature values per subject. Spatially constrained ICA is particularly advantageous for large multi-site datasets such as ABIDE, as it enforces spatial consistency while retaining the ability to identify spatially independent functional components.

The sICA procedure followed the standard multi-stage pipeline described previously [45,46]: (1) subject-level principal component analysis (PCA) was first applied individually to each participant’s data; (2) group-level PCA was then performed after temporal concatenation of subject-level data; (3) the spatially constrained ICA algorithm was applied to estimate ICNs; and (4) artifactual group-level components were identified and removed using established criteria [47].

This study aimed to identify network-level features associated with ASD that could facilitate network-based classification. The application of spatial constraint ICA enabled us to comprehensively identify brain functional connectivity signals, addressing the heterogeneity of ASD across diverse populations.

### 2.4. Network Selection and Component Pairing

Following preprocessing, spatially constrained ICA (scICA) was applied using the NeuroMark functional network template to decompose the brain into seven canonical resting-state networks: the Default Mode Network (DMN), Sensorimotor Network (SMN), Basal Ganglia Network (BGN), Auditory Network (AN), Attention Network (ATN), Visual Sensory Network (VSN), and Frontal Network (FN). These seven networks constitute a well-established, widely accepted functional parcellation in the neuroimaging literature [48]. The template further subdivides these networks into 28 predefined, anatomically and functionally validated components. Specifically, the DMN comprises four components (07, 17, 19, 26); the SMN includes six components (01, 05, 06, 08, 10, 21); the BGN and AN each consist of a single component (04 and 02, respectively); the ATN contains six components (09, 18, 20, 23, 27, 28); the VSN also includes six components (11, 13, 15, 22, 24, 25); and the FN is composed of four components (03, 12, 14, 16). Guided by neurofunctional relevance and prior evidence linking these networks to Autism Spectrum Disorder [49,50], the present study examined all 28 components across the seven standard resting-state networks to ensure that the network-level analyses were biologically grounded and reproducible.

Network selection was guided by three quantitative and neurobiologically grounded criteria. First, component quality and spatial correspondence were assessed by computing spatial correlations with the NeuroMark atlas templates (requiring r > 0.5) and applying standard GIFT toolbox quality metrics, including peak z-scores of spatial maps exceeding 3.0 and acceptable temporal signal-to-noise ratios. Second, neurobiological relevance to Autism Spectrum Disorder (ASD) was considered, with priority given to networks consistently implicated in prior ASD research, particularly the Default Mode Network (DMN), sensorimotor network (SMN), and visual sensory network (VSN), which are associated with social cognition, sensory integration, and motor processing, respectively. Third, to ensure robustness in multi-site analyses, only networks represented by at least four components consistently identified across all sites were retained.

This selection procedure is standard in ICA-based neuroimaging studies, where the interpretability of components is important. After selecting the networks and components, identical components from these networks were harmonized using the ComBat model across all sites.

### 2.5. Harmonization of fMRI Datasets

Combining neuroimaging data from multiple sites is often necessary to achieve suitable sample sizes and ensure consistency, providing reliable results [51,52]. However, prior studies have highlighted challenges in merging fMRI datasets due to variations in imaging techniques [53,54]. Despite efforts to standardize imaging procedures, inconsistencies persist due to factors such as scanner brand, magnetic field strength, coil type, stabilization mechanisms, channel numbers, image resolution, and pulse sequence settings (e.g., TR, TE, and flip angle) [55]. Specifically, ComBat harmonization [56] was performed using site as the batch variable, while age and sex were included as covariates to preserve biologically meaningful variance. This approach ensures that site-related effects are minimized while controlling for demographic variability. The harmonized features were subsequently used in the classification pipeline. The whole dataset (n = 996) was subjected to ComBat harmonization following ICA feature extraction but before cross-validation. This approach is suitable for ComBat because it needs sufficient within-site sample sizes to precisely calculate site specific mean and variance parameters. Importantly, site effects represent technical artifacts, which are constant for all subjects. ComBat removes all effects while maintaining biological signals. The parameters obtained from all the sites were used to harmonize the reserved test site for leave-one-site-out cross-validation. Although the test site is included in parameter estimations, this does not constitute information leakage in the traditional sense because the harmonization is only informed by site-level technical factors instead of individual subject data. This approach is useful for harmonizing test sites using only training site parameters, which would apply corrections obtained from different scanner types. Additionally, every machine learning step that followed LDA and PCA hyperparameters adjustment and SVM training was caried out only inside training folds with strict test isolation. This ensures that only training data is used for test prediction [57], while harmonization simply takes care of technical variance. The ComBat harmonization model and mathematical formulations were explained in Section S1 of the Supplementary Materials [58].

### 2.6. Feature Extraction and Selection

Spatial features were derived by extracting voxel-wise z-score-normalized intensities from each component’s spatial map. After applying a group-level brain mask, each component contained approximately 214 voxels. Across 28 components, voxel intensities were vectorized, yielding approximately 6000 spatial features per subject across the selected networks.

Selecting the right features is important, mainly when using large feature vectors. A high feature-space can decrease accuracy, increase training and testing times, and increase model complexity. Features selection decreases feature size, improves model generalizability, and increases classification performance [59,60,61]. A two-step dimensionality reduction approach was used to handle high feature dimensionality and reduce redundancy. Principal Component Analysis (PCA) and Linear Discriminant Analysis (LDA) were used to minimize high dimensionality. PCA keeps the most important features, whereas LDA improves the selection to maximize class separation [62]. PCA was only applied to training folds (not to test data) to avoid data leakage. PCA-transformed data was used as an input to LDA. PCA and LDA were explained in Section S2 of the Supplementary Materials [58].

### 2.7. SVM as a Classification Model

The classifier uses the information given to it to build a hyperplane between distinct groups, such as ASD and HC, in a space with several characteristics. The SVM finds the best separating hyperplane between two classes while maintaining the maximum distance between them. SVM classification was performed using the radial basis function (RBF) kernel. Model parameters were optimized through grid-search tuning and the best parameter pairs (b*, y*) were selected based on the average cross-validation area under the curve (AUC). In all experiments, cross-validation was implemented using site-specific data splits to ensure balanced representation of sites in both training and testing sets, thereby mitigating site-related bias. Classification analyses were conducted at two levels: (1) within individual sites to assess site-specific performance, and (2) across combined sites to evaluate model generalizability under diverse acquisition settings. This dual-level approach enhances the robustness and external validity of the classification results. Dimensionality reduction steps were repeated independently for each site and network, confirming that no data leakage occurred. Mathematically, it can be expressed in Section S3 of the Supplementary Materials [58].

### 2.8. Evaluation

To validate the efficiency of the proposed ASD/HC classification approach, several performance indices were calculated, namely accuracy (ACC), area under the curve (AUC), sensitivity (SEN), and specificity (SPE) [63,64]. The performance metrics can be defined mathematically and are provided in Section S4 of the Supplementary Materials [58].

### 2.9. Implementation

All the analyses were performed in MATLAB (version R2022b; The MathWorks Inc., Natick, MA, USA) using GIFT toolbox (Group ICA of fMRI Toolbox, version 4.0.4.11; www.nitrc.org/projects/gift; The Mind Research Network, Albuquerque, NM, USA), DPARSFA toolbox (version V7.0; http://www.rfmri.org; State Key Laboratory of Cognitive Neuroscience and Learning, Beijing Normal University, Beijing, China). Computation was performed on a workstation intel i9-13900k CPU (Intel Corporation, Santa Clara, CA, USA), 64 GB RAM, RTX 4090 GPU, Windows 11.

## 3. Results

**Figure 1:**
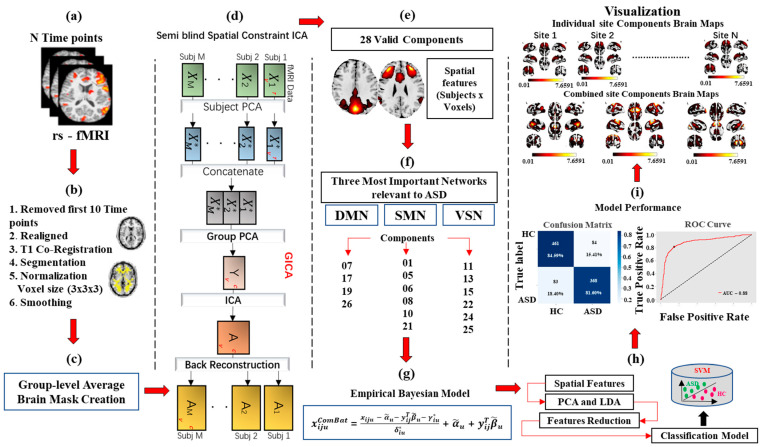
Overview of the schematic pipeline processing method including (**a**) rs-fMRI data acquisition; (**b**) preprocessing; (**c**) group mask generation; (**d**) spatial constraint ICA; (**e**) feature extraction; (**f**) network grouping; (**g**) ComBat harmonization; (**h**) feature selection and classification; (**i**) final visualization.

The demographic information of the cumulative records of 996 subjects is shown in Table 1. The detailed information of global diagnostic imaging sites for Autism Spectrum Disorder from the ABIDE library are shown in Supplementary Material Table S1 [58]. Table S2 of the Supplementary Material [58] contains details of the institutions and the scanner selection parameters and all the experimental settings of all 11 sites. The detailed research methodology employed in this study is outlined in Figure 1, which presents a unique workflow.

### 3.1. Classification Results

The DMN, SMN, and VSN satisfied all criteria and were therefore selected for downstream analyses. Other networks were excluded for objective reasons: the basal ganglia network (BGN) and auditory network (AN) contained only single components; the attention network (ATN) showed a substantially higher mean artifact component ratio (0.23 vs. 0.08 in selected networks); and the frontal network (FN) demonstrated lower mean spatial correlation across sites (0.52 compared to 0.71 in selected networks), indicating reduced cross-site consistency. These clarifications are explicitly stated in the Section 2 prior to harmonization.

In the classification results, individual-site and group-level site analysis shows outstanding results for ASD and HC classification, with individual-site analysis achieving up to 80.40% pointing out spatial features. Multi-site analysis showed accuracy of up to 83.23%.

#### 3.1.1. Individual-Site Classification Results

To determine the versatility of the proposed networks analysis approach, we present the results of the top three brain networks associated with ASD and HC classification. The highest accuracies for DMN, SMN, and VSN are 80.40%, 79.72%, and 80.40%, with specificities of 87.87%, 88.54%, and 90.32% and sensitivities of 65.30%, 63.46%, and 63.63%. The AUCs were 79.54%, 82.97%, and 83.17%. See Table 2 for detailed results across sites and networks.

Table 2 presents the classification results of three main networks (DMN, SMN, and VSM) across multiple sites’ components, calculating accuracy (ACC), area under the curve (AUC), specificity (SP), and sensitivity (SN). The VSM frequently achieves higher accuracy and specificity, representing a stronger performance. The DMN and SMN show a little variability across sites, with some occurrences of higher sensitivity. This shows site-dependent differences in network effectiveness for precise classification in this context.

**Figure 2:**
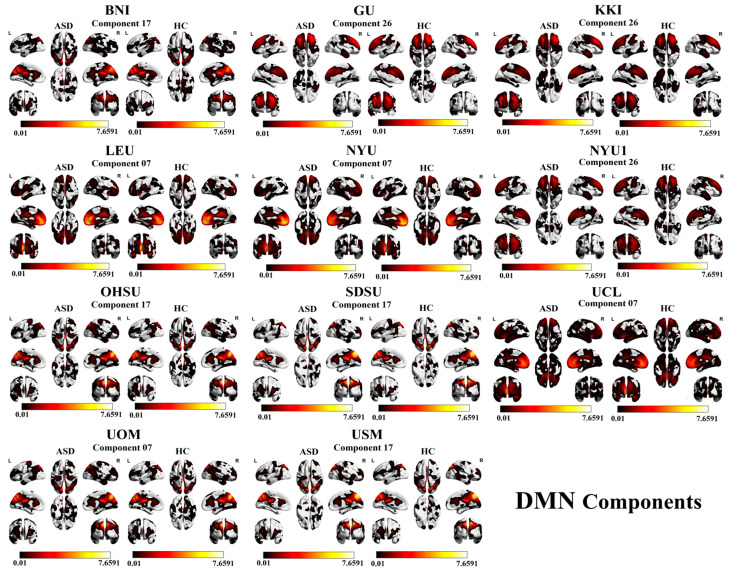
The highest classification accuracy across 11 independent sites, grouped as posterior vs. anterior. ASD and HC activation maps are visualized as axial slices, with color intensity reflecting the strength of activation.

**Figure 3:**
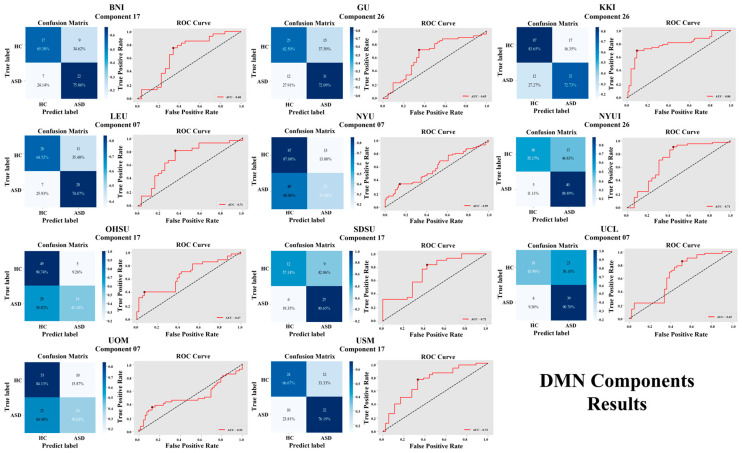
Classification performance of DMN components across 11 independent sites, showing confusion matrices and ROC curves for four ICA components (07, 17, 19, 26).

For the DMN, the individual sites that show high accuracies are as follows: BNI with component 17 (70.91%), GU with component 26 (67.47%), KKI with component 26 (80.41%), LEU with component 07 (68.97%), NYU with component 07 (63.95%), NYU1 with component 26 (75.68%), OHSU with component 17 (71.59%), SDSU with component 17 (71.15%), UCL with component 07 (67.86%), UOM with component 07 (66.35%), and USM with component 17 (71.79%). A visual representation of the DMN component results is shown in Figure 2 and Figure 3.

**Figure 4:**
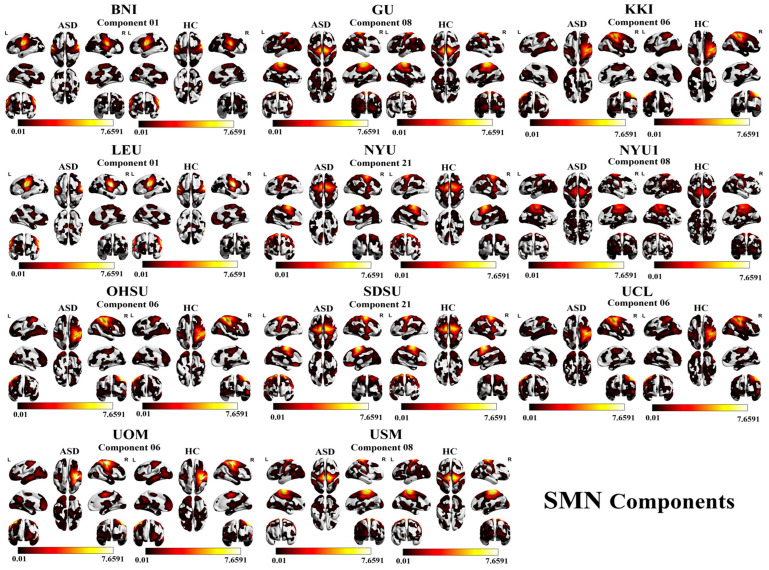
SMN had the highest classification accuracy across 11 independent sites. Activation patterns involve regions within the postcentral gyrus, superior frontal gyrus, and paracentral lobule. Spatial maps are shown for ASD and HC groups, with each row representing one site and axial slices visualizing activation intensity.

**Figure 5:**
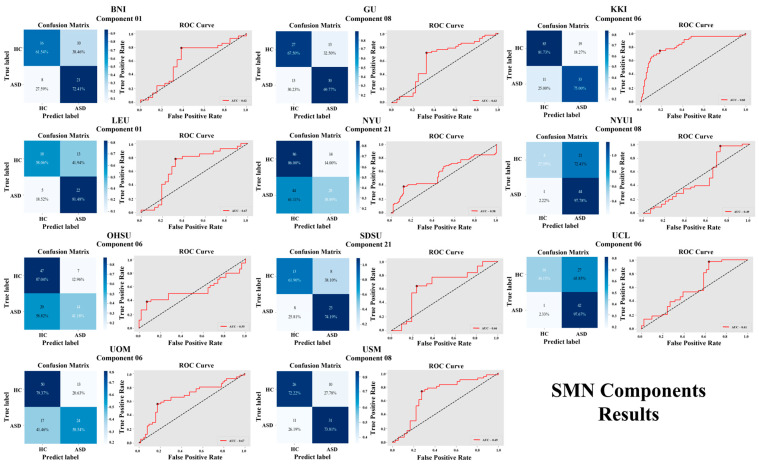
Classification performance of SMN components across 11 independent sites, showing confusion matrices and ROC curves for six ICA components (01, 05, 06, 08, 10, and 21).

The accuracy results for the SMN are as follows: BNI with component 06 (67.27%), GU with component 08 (68.67%), KKI with component 06 (79.73%), LEU with component 01 (68.97%), NYU with component 21 (66.28%), NYU1 with component 08 (70.27%), OHSU with component 06 (69.32%), SDSU with component 21 (69.23%), UCL with component 06 (66.67%), UOM with component 05 (71.15%), and USM with component 08 (73.08%). A visual representation of SMN component results is shown in Figure 4 and Figure 5.

**Figure 6:**
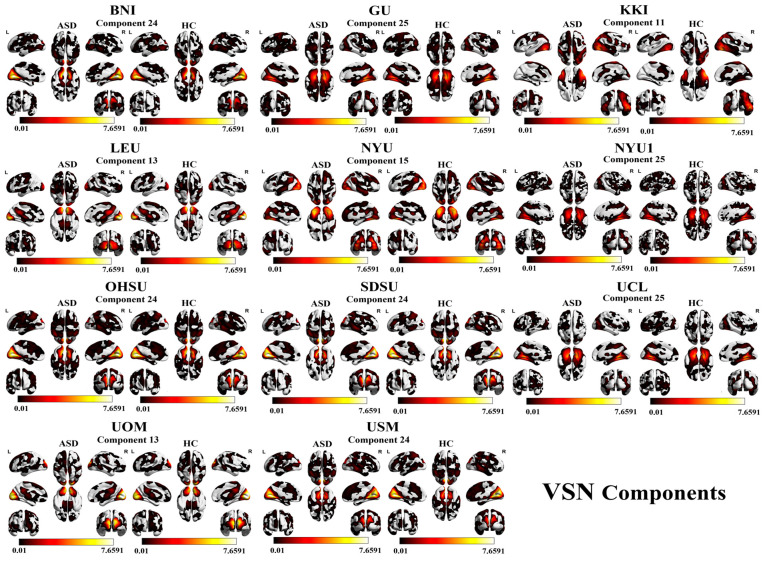
The highest classification accuracy across 11 independent sites, showing site-specific activation patterns in occipital, fusiform, temporal, and cerebellar regions. Each row displays ASD and HC axial activation maps, with color intensity indicating activation strength.

**Figure 7:**
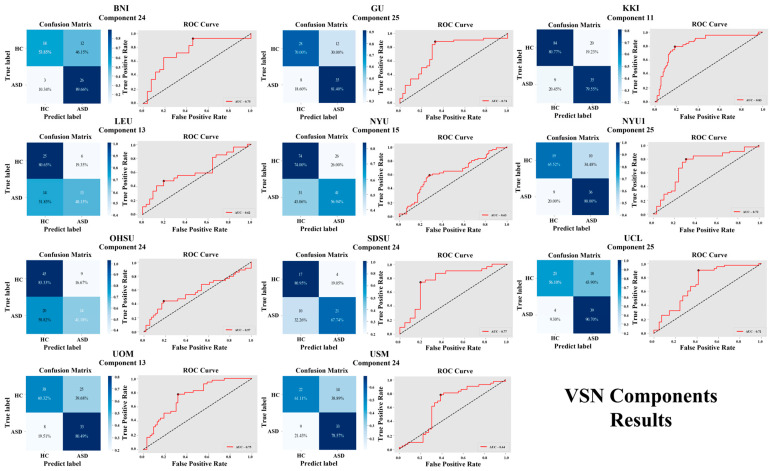
Classification performance of VSN components across 11 independent sites, showing confusion matrices and ROC curves for six ICA components (11, 13, 15, 22, 24, 25).

For the VSN, the components showing high accuracy for all the sites are as follows: BNI with component 24 (72.73%), GU with component 25 (75.90%), KKI with component 11 (80.41%), LEU with component 13 (65.51%), NYU with component 13 (66.86%), NYU1 with component 25 (74.32%), OHSU with component 24 (67.04%), SDSU with component 24 (73.07%), UCL with component 25 (73.80%), UOM with component 13 (68.26%), and USM with component 24 (70.51%). A visual representation of SMN component results is shown in Figure 6 and Figure 7.

Spatial features were used for individual sites (see Supplementary Tables S3.1–S3.3 in the Supplementary Materials and Supplementary Figures S1–S10) for full results of the individual components [58].

#### 3.1.2. Multi-Site Combined Results

This investigation applied the current proposed network analysis approach to the multi-site ASD data from 11 sites of ABIDE-I and II for classification. Spatial features were used as an input. Due to known inter-site heterogeneity factors like age and gender, the ComBat harmonization technique was applied as described above. Table 3 presents all three network results of accuracy (ACC), sensitivity (SN), specificity (SP), and area under the curve (AUC).

Higher sensitivity was observed in the combined multi-site analyses compared with individual-site results, reflecting the effects of increased sample size, harmonization, and improved classifier stability, rather than simply aggregating site-level performance metrics. The performance measures reported in Table 3 differ from those in Table 2 due to differences in the feature representation and preprocessing strategy. In the combined multi-site analysis, features were harmonized using ComBat and represented as mean voxel intensities within component masks, whereas individual-site analyses (Table 2) relied on voxel-level spatial features without harmonization. Harmonized features are less sensitive to scanner- and site-specific variability and reduce the risk of overfitting when pooling heterogeneous multi-site data. In contrast, voxel-level features preserve fine-grained spatial information, which is advantageous for single-site analyses with uniform acquisition parameters but may limit generalizability across sites.

Additionally, pooling data across sites substantially increases the effective sample size and the diversity of training examples, enabling the classifier to learn more stable, generalized decision boundaries. The combination of harmonization increased subject diversity and reduced technical variance, which explains why the sensitivity in the combined analysis exceeded that observed in individual-site analyses, rather than reflecting an inconsistent model performance.

Table 3 shows that for DMN, SMN and VSN, the model achieved 81.43%, 80.52%, and 83.23% highest accuracies, respectively. The specificities were as follows: 82.14%, 83.43%, and 84.74%, with sensitivities of 80.50%, 77.28%, and 81.42%. The AUC of all the networks was 84.53%, 84.96%, and 87.90%. The current approach addresses inter-site heterogeneity using harmonization and minimizes feature variations between sites, whereas other approaches neglect heterogeneity when merging multi-site data. These outcomes support the importance of standardizing rs-fMRI data in multi-site research.

**Figure 8:**
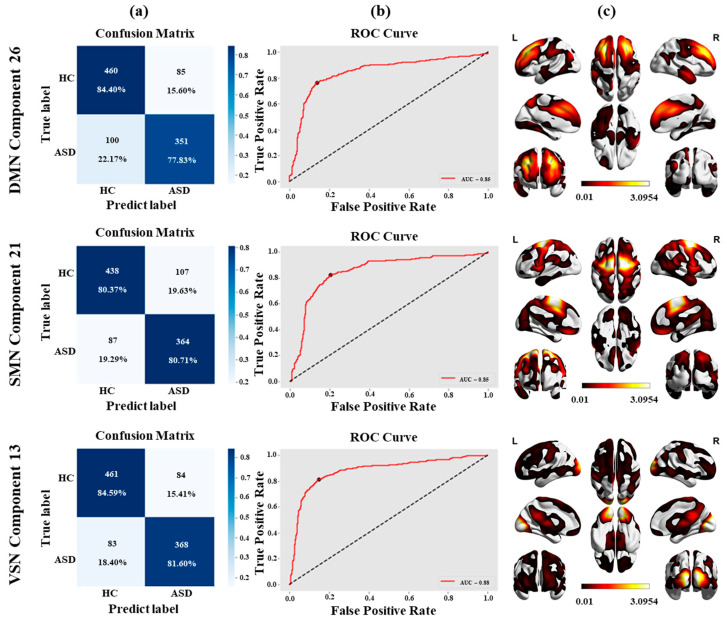
Classification performance and spatial visualization for DMN, SMN, and VSN. Column (**a**) shows confusion matrices; column (**b**) presents ROC curves with AUC values; and column (**c**) displays averaged spatial maps across all sites.

Figure 8 shows all these results. Table 3 and Figure 8 show that the proposed network analysis approach delivers the most favorable results across all 11 sites (see Supplementary Figures S11–S13 for full results) [58]. For combined sites, the VSN achieved the highest performance (ACC: 83.23%, AUC: 87.90%, SP: 84.74%, SN: 81.42%).

This proposed model’s performance is compared with prior ASD classification studies to investigate these results. The obtained classification metrics demonstrate the effectiveness of the current model across selected network components. A detailed comparative analysis with existing studies is discussed in Table 4.

Table 4 presents a qualitative comparison based on previous studies for context. However, because of significant methodological variability, direct performance comparisons need to be interpreted with care, as different ABIDE datasets may include multiple sites and subjects, distinct preprocessing pipelines, multiple validation approaches (some using single-site validation), and different feature extraction methods. As a result, the current findings present well-established methods that provide significant advantages in multi-site consistency. This research not only provides classification accuracy but also supports a multi-site harmonization approach, network-specific interpretability, and a reproducible analysis framework.

## 4. Discussion

Past studies have investigated different aspects of brain connectivity in ASD. Unlike those that usually relied on small datasets, our research used a large dataset of 996 individuals from 11 independent sites and achieved higher classification accuracy using SVM. The novelty of this research lies not only in its use of multi-site datasets but in its creation of a harmonization-validated, network-interpretable, computationally explainable framework. Furthermore, instead of only addressing the performance metrics, this study conducts a difficult heterogeneity and constancy analysis, recognizing the visual networks as a reproducible biological signature.

Current findings align with and expand upon previous studies that highlight the DMN, SMN, and VSN as critical networks in understanding the neural underpinnings of ASD. There is strong theoretical and empirical support for the selection of these networks and components. The DMN is fundamentally involved in self-referential thinking, social cognition, and introspection—processes that are frequently impaired in individuals with ASD [81,82]. The altered connectivity within the DMN may contribute to the social alterations observed in ASD, as individuals often struggle with understanding social cues, theory of mind, and engaging in typical social interactions [83,84]. Also, these alterations in the DMN are thought to reflect difficulties in shifting attention between internal and external stimuli, which may underlie the social and communication challenges characteristic of ASD.

The SMN, on the other hand, plays a pivotal role in motor regulation and sensory processing, both of which are often compromised in people with ASD. The motor and sensory processing differences common in people with ASD suggest a direct link between the SMN function [82,85]. For instance, motor challenges may affect nonverbal communication, such as gestures and facial expressions, while sensory sensitivities may lead to avoidance or overreaction to environmental stimuli.

In this study, the VSN emerged as the most important feature for classification accuracy when it came to distinguishing individuals with ASD from controls. This finding highlights the VSN’s critical role in the pathophysiology of ASD, particularly in relation to sensory processing. Previous research has established that individuals with ASD often exhibit atypical sensory processing, which aligns with the significant contributions of the VSN to sensory integration and altered brain connectivity [86,87]. The VSN’s involvement in integrating visual inputs and connecting sensory information is essential for understanding how individuals with ASD perceive and interact with their environment. For example, altered VSN connectivity may contribute to the sensory overload commonly addressed in ASD, where individuals experience heightened sensitivity to visual stimuli, leading to difficulties filtering out irrelevant information and focusing on socially relevant cues.

The importance of the DMN, SMN, and VSN suggests that network-level interconnections are key ASD symptoms. For instance, the DMN’s role in self-referential thought processes can influence how sensory information is interpreted and integrated by the SMN and VSN [82,88]. The VSN’s role in processing visual information is particularly relevant, as it has been implicated in the sensory overload that many individuals with ASD experience. This sensory overload can exacerbate social communication difficulties, as individuals may become preoccupied with sensory inputs rather than engaging with social cues. Outside of these networks, components often showed lower stability or were affected by noise and physiological artifacts, which would reduce interpretability and compromise classifier robustness.

Furthermore, to address the heterogeneity and variability in neuroimaging data, this study demonstrated that by combining spatial constraint ICA with ComBat harmonization provides a robust and reproducible technique for handling multi-site resting state data by using spatial priors. Spatial constraint ICA ensured component-level correspondence across subjects and sites by standardizing the extracted networks before harmonization. The authors of [43] discuss how spatial constraints can enhance the identification of commonly occurring networks, such as the DMN, across different subjects and tasks. The ComBat model further reduced the scanner variability and stabilized the feature distribution across all 11 sites, thereby enhancing the generalizability and reliability and confirming the effectiveness of this pipeline. The combination of these techniques enabled us to achieve high classification accuracy by justifying inter-site differences and providing a positive answer to the first research question.

All the results presented in this paper show that specific functional brain networks provided meaningful discriminative information for classifying ASD from healthy controls. These findings suggests that interventions targeting the DMN, SMN, and VSN may hold promise for supporting individuals with core features of ASD. For example, neurofeedback or brain-stimulation techniques aimed at modulating connectivity within these networks could potentially improve social cognition, sensory processing, and motor coordination in individuals with ASD. Additionally, behavioral interventions that focus on sensory integration and social skills training may benefit from incorporating strategies that address the specific neural mechanisms underlying these challenges. All these findings show that network-level features extracted from spatial constraint ICA not only capture meaningful neurobiological differences but also preserve discriminative control across heterogeneous imaging sites, addressing our second research question.

Dimensionality reduction of features using LDA/PCA with the SVM classifier resulted in a robust performance across networks and sites. The multi-site SVM classifier was trained on harmonized features, highlighting the importance of combining large-scale multi-site data. In particular, the VSN yielded an accuracy of 83.23%, emphasizing the critical role of harmonizing data from diverse sources. A network-specific comparison also revealed statistically meaningful differences in performance. The proposed approach performed significantly better in ASD classification than other existing methods and the findings directly support third research question by showing the harmonized network features and effective machine learning-based classification in a multi-site context. However, there are specific constraints to consider.

This study emphasizes that the results are derived from ABIDE data with standardized diagnostic procedures and quality control, and that external validation on independent cohorts, including different institutions, scanner manufacturers, demographic compositions, and geographic regions, is required before any translational claims can be considered. This research also explicitly acknowledges persistent challenges in rs-fMRI-based ASD classification, including inter-site variability, limited reproducibility, and developmental effects. Consistent with prior work [89], it is stated that substantial methodological and validation hurdles must be tackled before neuroimaging-based classification can demonstrate added value beyond established behavioral assessments. Accordingly, the revised manuscript positions the present work as a methodological framework for identifying network-level patterns relevant to ASD neurobiology rather than as an immediate clinical diagnostic tool. Though we used data from 11 different institutions, the absence of individual data gathering is a significant limitation. The results of the present study are dependent solely on the ABIDE I and II datasets, which could be insufficient. High-dimensional features may still result in bias even when SVM reduces overfitting through normalization. However, this research employed cross-validation to avoid leakage and PCA and LDA to reduce dimensionality.

Future research should integrate additional ASD clinical datasets from various sources, preferably across different age groups, cultural contexts, and MRI acquisition protocols, to strengthen the assessment of the classification method and generalizability beyond ABIDE and help us to evaluate the model’s robustness in a range of context rather than performing evaluations based solely on past research datasets. While this study identified which large-scale brain networks and associated spatial regions contribute to group classification, it did not examine the relationship between these network-level features and core clinical dimensions of Autism Spectrum Disorder (ASD), such as social communication deficits and restricted, repetitive behaviors, as measured by standardized instruments like the ADOS and ADI-R. Furthermore, the potential of these network markers to track longitudinal symptom trajectories remains unexplored. Addressing these gaps in future research is essential to bridge the divide between neuroimaging-based classification and clinically meaningful outcomes, ultimately advancing the translational utility of functional connectivity biomarkers in ASD. The observed differences in classification performance across functional networks were relatively modest and were reported descriptively. No formal statistical comparison, such as parametric or non-parametric hypothesis testing or confidence interval estimation, was performed to assess the significance of these differences. Future studies should incorporate rigorous statistical frameworks to quantitatively evaluate and compare the discriminative power of individual networks, thereby strengthening the interpretability and reliability of network-based biomarkers in ASD classification. Furthermore, existing research excluded two additional MRI modalities (structural MRI and diffusion tensor imaging). The use of various modalities could enhance classification by offering more information. Also, investigating the predictive ability of the current machine learning framework could uncover the important functional correlations connected to ASD processes. Although these drawbacks were not taken into account in this investigation, we want to consider them in future ASD identification research. In future work, we will collaborate with neurologists and radiologists to qualitatively evaluate components’ relevance and assess the potential of the proposed framework.

## 5. Conclusions

The proposed brain network analysis approach produced promising results across all 11 single and combined sites. These findings point out the critical roles of the DMN, SMN, and VSN in the functional connectivity architecture of ASD, with the VSN evolving as the most important network for classification. The use of spatially constrained ICA and ComBat shows the importance of strong signal processing and data harmonization approaches in neuroimaging research, particularly when it comes to addressing the site-specific heterogeneity and data variability that are common in multi-site rs-fMRI studies. These findings not only develop our understanding of the neural foundations of ASD but also provide a basis for targeting the brain networks involved in ASD disorder. Future research should continue to explore the dynamic interactions between these networks and their potential as biomarkers for identification and behavior response in ASD.

## Figures and Tables

**Table 1:** Cumulative record of ASD datasets for ABIDE-I and ABIDE-II repositories.

Repositories	Sites	ASD Male	ASD Female	HC Male	HC Female
ABIDE-I	05	209	16	235	36
ABIDE-II	06	182	44	225	49
Total	11	391	60	460	85

Note: ABIDE I and II: The Autism Brain Imaging Data Exchange.

**Table 2:** Individual sites’ classification results for all three networks, DMN, SMN, and VSN, using voxel-level spatial features showing the highest-performing components of each site.

Sites	Networks	Component Numbers	Accuracy (%)	AUC (%)	Specificity (%)	Sensitivity (%)
BNI	DMN	17	70.91%	67.64%	70.83%	70.97%
	SMN	06	67.27%	69.63%	64.29%	70.37%
	VSM	24	72.73%	74.80%	82.35%	68.42%
GU	DMN	26	67.47%	65.12%	67.57%	67.39%
	SMN	08	68.67%	62.73%	67.50%	69.77%
	VSM	25	75.9%	74.36%	77.78%	74.47%
KKI	DMN	26	80.41%	79.55%	87.88%	65.31%
	SMN	06	79.73%	82.98%	88.54%	63.46%
	VSM	11	80.41%	83.17%	90.32%	63.64%
LEU	DMN	07	68.97%	71.21%	74.07%	64.52%
	SMN	01	68.97%	67.14%	78.26%	62.86%
	VSM	13	65.51%	62.37%	64.10%	68.42%
NYU	DMN	07	63.95%	58.83%	63.97%	63.89%
	SMN	21	66.28%	58.49%	66.15%	66.67%
	VSM	13	66.86%	64.75%	67.48%	65.31%
NYU1	DMN	26	75.68%	71.03%	76.19%	75.47%
	SMN	08	70.27%	48.97%	88.89%	67.69%
	VSM	25	74.32%	71.80%	67.86%	78.26%
OHSU	DMN	17	71.59%	66.50%	71.01%	73.68%
	SMN	06	69.32%	55.01%	70.15%	66.67%
	VSM	24	67.04%	57.46%	69.23%	60.87%
SDSU	DMN	17	71.15%	72.35%	66.67%	73.53%
	SMN	21	69.23%	65.59%	61.90%	74.19%
	VSM	24	73.07%	76.65%	62.96%	84%
UCL	DMN	07	67.86%	65.34%	81.82%	62.90%
	SMN	06	66.67%	60.81%	93.33%	60.87%
	VSM	25	73.8%	71.81%	85.19%	68.42%
UOM	DMN	07	66.35%	52.65%	67.95%	61.54%
	SMN	05	71.15%	74.29%	85.11%	59.65%
	VSM	13	68.26%	74.72%	82.61%	56.90%
USM	DMN	17	71.79%	73.15%	70.59%	72.73%
	SMN	08	73.08%	69.18%	70.27%	75.61%
	VSM	24	70.51%	63.62%	70.97%	70.21%

**Table 3:** Multi-site combined classification results for all the network components using spatial harmonized features.

Networks	Component Numbers	Accuracy (%)	AUC (%)	Specificity (%)	Sensitivity (%)
DMN	07	80.12%	83.01%	82.92%	76.97%
	17	79.82%	83.87%	83.59%	75.83%
	19	78.01%	83.08%	82.86%	73.20%
	26	81.43%	84.53%	82.14%	80.50%
SMN	01	78.11%	82.40%	82.00%	74.02%
	05	79.42%	83.95%	83.46%	75.20%
	06	78.21%	81.70%	80.48%	75.55%
	08	79.92%	85.53%	83.63%	75.98%
	10	80.12%	84.62%	83.17%	76.74%
	21	80.52%	84.96%	83.43%	77.28%
VSN	11	80.32%	85.82%	82.26%	78.02%
	13	83.23%	87.90%	84.74%	81.42%
	15	78.11%	82.64%	81.38%	74.53%
	22	80.42%	84.88%	83.78%	76.78%
	24	80.22%	84.77%	82.34%	77.73%
	25	82.93%	87.32%	84.03%	81.57%

**Table 4:** The results comparison of machine learning-based ASD classification and identification approaches using ABIDE data.

References	Year	Samples ASD/HC	Features	Classifier	ACC (%)	SN (%)	SP (%)
Proposed	2025	451/545	Spatial Constrained ICA	SVM	83.23	81.42	84.74
[23]	2020	45/47	D-FCNs	SVM	83.00	82.00	84.00
[65]	2025	398/397	LeNet5	MLP	82.30	U/N	U/N
[66]	2021	193/292	Statistical Analysis	Sparse LR	82.14	79.70	83.74
[67]	2023	115/106	LSTM	ANN	80.00	81.00	80.00
[68]	2021	403/468	Power264	DNN	79.20	U/N	U/N
[20]	2020	24/35	AAL	SVM	79.00	U/N	U/N
[24]	2020	403/468	DFC	MTFC	76.80	72.50	79.90
[69]	2021	201/251	AAL, CPAC	DT	75.00	U/N	U/N
[25]	2020	419/530	AAL, Dosenbach, CC200	MLP + Ensemble Learning	74.52	80.69	66.71
[70]	2020	399/472	CC200, CPAC	SVM, KNN, LDA, Ensemble Trees	72.50	94.00	64.70
[22]	2025	525/532	Pair-wise PCC	CNN	72.42	71.68	72.73
[21]	2021	79/105	FNCs	No Super Parameter FCN	71.74	65.82	76.19
[71]	2021	159/184	U/N	Three-way decision model	71.35	82.35	61.52
[26]	2021	408/476	EZ, HO, TT, CC200, AAL, Dosenbach160	SVC	71.10	66.00	75.60
[72]	2023	505/530	CC200	AE, DiagNet + SLP, AE	70.90	70.70	75.50
[73]	2022	280/329	BASC64	SVD, SVM, MC-NFE	68.42	70.05	63.64
[74]	2021	306/350	BASC333	RF	67.81	60.00	65.00
[75]	2020	432/556	CC200	RCE-SVM	67.30	64.50	70.10
[76]	2020	403/468	GFT	RBF-SVC	66.70	62.38	72.35
[77]	2020	245/272	NAG-FS	SVM	65.03	U/N	U/N
[78]	2020	505/530	CC200	AE-MKFC	61.00	U/N	U/N
[79]	2021	103/192	ICA, IBMA	RBF-SVM	59.70	48.40	71.00
[80]	2020	493/542	CC200	NEG + MLP	58.70	61.50	56.90

Note: ACC = accuracy; SN = sensitivity; SP = specificity; ASD = autism spectrum disorder; HC = healthy control; U/N = unknown; SVM/SVC = support vector machine; LDA = linear discriminant analysis; RF = Random Forest; NEG = negative correlation matrix; FCN = functional connectivity network; DFC = dynamic functional connectivity network; RBF = radial basis function; MLP = multilayer perceptron; RCE = recursive cluster removal.

## Data Availability

The resting-state fMRI data used in this study are publicly available from the Autism Brain Imaging Data Exchange (ABIDE) repository at http://fcon_1000.projects.nitrc.org/indi/abide/ (accessed on 15 August 2025). This dataset includes contributions from 38 different sites worldwide, encompassing a total of 2264 participants, comprising 1083 subjects diagnosed with ASD and 1181 healthy controls. The analysis scripts used in this study, including classification and ComBat harmonization codes, are publicly available at the following GitHub repository: https://github.com/TalhaImtiaz/SVC-and-ComBat-Scripts (accessed on 5 September 2025).

## Supplementary Materials

The following supporting information can be downloaded at: https://www.mdpi.com/article/10.3390/brainsci16020181/s1, Table S1: Global diagnostic imaging sites for Autism Spectrum Disorder from the ABIDE library were used in this study; Table S2: Scanner acquisition parameters and experimental settings of 11 sites from ABIDE-I and II; Table S3.1: Individual site classification results of all the components of the DMN; Table S3.2: Individual site classification results of all the components of the SMN; Table S3.3: Individual site classification results of all the components of the VSN; Figure S1: DMN components (07, 17, 19, 26) across 11 independent sites; Figure S2: Classification performance of DMN ICA components (07, 17, 19, 26) across 11 independent sites; Figure S3: Visualization of SMN components (01, 05, 06) across 11 independent sites; Figure S4: Visualization of SMN components (08, 10, 21) across 11 independent sites; Figure S5: Classification performance of SMN ICA components (01, 05, 06) across 11 independent sites; Figure S6: Classification performance of SMN ICA components (08, 10, 21) across 11 independent sites; Figure S7: Visualization of VSN components (11, 13, 15) across 11 independent sites; Figure S8: Visualization of VSN components (22, 24, 25) across 11 independent sites; Figure S9: Classification performance of VSN ICA components (11, 13, 15) across 11 independent sites; Figure S10: Classification performance of VSN ICA components (22, 24, 25) across 11 independent sites; Figure S11: Visualization of the average spatial brain maps of DMN network components (07, 17, 19, 26); Figure S12: Visualization of the average spatial brain maps of SMN network components (01, 05, 06, 08, 10, 21); Figure S13: Visualization of the average spatial brain maps of VSN components (11, 13, 15, 22, 24, 25).
