# Supplementary material for: Multi-Site Classification of Autism Spectrum Disorder Using Spatially Constrained ICA on Resting-State fMRI Networks
Source: Brain Sci. 2026 Jan 31;16(2):181. doi: 10.3390/brainsci16020181 (PMC12938527; doi:10.3390/brainsci16020181)
Supplement: Supplementary file 1 [file brainsci-16-00181-s001.zip › brainsci-4054772-supplementary.pdf]

Supplementary Materials

Multi-Site Classification of Autism Spectrum Disorder Using Spatially Constrained ICA on Resting-State fMRI Networks

S1.Harmonization of fMRI Datasets

ComBat's harmonization capabilities address potential biases arising from differences in imaging equipment and demographic factors like age and gender. The algorithm normalizes features across sites, maintaining consistency without altering relevant biological signals.

First, we normalize the data at each site by subtracting the mean value of the site and then dividing by its standard deviation for each feature (e.g., voxel density). Combat hypothesizes that the data follows a certain pattern. The raw data for a given feature, denoted as x_{iju}, is modeled using the equation: (1):

$$\mathrm{x_{iju}} = \alpha_{\mathrm{u}} + \mathrm{y}_{\mathrm{ij}}^{\mathrm{T}}\beta_{\mathrm{u}} + \gamma_{\mathrm{iu}} + \delta_{\mathrm{iu}}\mathrm{e_{iju}} \tag{1}$$

Here, $\mathrm{x_{iju}}$ represents the observed data for subject i at site j during measurement u. The parameter a_u serves as the baseline intercept across all subjects and sites, while y_{ij}^T denotes covariates for the feature, and β_u, indicates the coefficients reflecting covariate effects. Site-specific effects are captured by γ_{iu} (mean) and δ_{iu} (variance), with e_{iju} representing the residual error, assumed to follow a normal distribution ~ N (0, σ_u^2).

In the first step, the least squares estimate of $\tilde{\alpha}_u$ is the baseline of features across all individuals and sites whenever other variables are zero and $\tilde{\beta}_u$ represents the covariate effects on measurements and also shows whether ASD and HC have any effects on brain measurements.

Combat then proceeds to assume that site effects have an identical distribution for all features, that the mean of site effects γ_{iu} follows an independent regular distribution from subject i at site j during u, and that the variance of site effects δ_{iu} regulates the heterogeneity in the site effect between subjects within the same site.

Hyperparameters are estimated from the data for all features using the method of moments. Bayesian empirical estimates of the resulting points γ_{iu}^* and δ_{iu}^* are obtained as mean values of the posterior distribution. Finally, the harmonized ComBat data are calculated from these estimates using Equation (2):

$$x_{\mathrm{i}ju}^{ComBat} = \frac{x_{iju} - \tilde{\alpha}_u - y_{ij}^T\tilde{\beta}_u - \gamma_{\mathrm{iu}}^*}{\delta_{\mathrm{iu}}^*} + \tilde{\alpha}_u + y_{ij}^T\tilde{\beta}_u \tag{2}$$

The main objective of Combat is thus, to estimate the coefficients, taking into account the effects of the sites. Since its release and proven effectiveness, Combat has been used and recognized widely in MRI studies

S2.Feature selection

For feature selection, the combination of PCA and LDA proved to be highly effective. By giving priority to important features, PCA firstly lowered the dataset size. LDA then improved this selection, ensuring that reliable data was kept for class differentiation. By optimizing the data and expanding the classifier's potential to distinguish across classes, these models together improved classification performance. In terms of mathematics,

PCA uses the following equation (3) to compute the covariance matrix C of the original data X and to find the eigenvalues (λi) and eigenvectors (vi):

$$C = \frac{1}{n} X^T X \tag{3}$$

Equation (4) was used to create the transformed data (Y) after the original data was mapped into eigenvectors. The most significant k eigenvectors are those that correspond to the biggest eigenvalues V.

$$Y = XV \tag{4}$$

Finding a projection matrix (W) that minimizes variation within classes (SW) and optimizes separation (SB) across classes is the main objective of LDA. Equation (5), which represents the eigenvalue problem, must be solved in order to achieve this.

$$S_W^{-1} S_B W = \lambda W \tag{5}$$

Equation (6) describes the data transformation process that occurs when PCA and LDA are coupled.

$$Z = YW \tag{6}$$

Z represents the features that have been selected for classification. By reducing redundancy and preserving the most useful information, these features eventually improve classification accuracy.

S3.SVM as a Classification Model

Mathematically, it can be expressed using Equation (7):

$$f(y) = \omega^T y + b \tag{7}$$

Here, $f(y)$ is the decision function and y is the input feature vector. Where ω indicates the value of the weight vector and b signifies the bias. Bias b modifies the hyperplane's orientation about its location in the space of features, while ω impacting its orientation. The function $f(y)$ has the goal of dividing sample sizes into two different categories. For this purpose, it gives a positive value to samples in one group and a negative value to samples in the second.

S4.Evaluation

Accuracy is the percentage of accurate predictions produced by the classifier in comparison with the true value during validation. In essence, it is the ratio of correct estimates to the overall count of made predictions. To calculate accuracy, follow Equation (8):

$$Accuracy = \frac{TN+TP}{TN+FN+TP+FP} * 100\% \tag{8}$$

The classifier correctly determines the percentage of positives recognized in the test phase, which is calculated using Equation (9):

$$Sensitivity = \frac{TP}{TP+FN} * 100\% \tag{9}$$

In our tests, the classifier effectively identified the percentage of true negatives, defined by the following Equation (10):

$$Specificity = \frac{FN}{TN+FP} * 100\% \tag{10}$$

Here TP, TN, FP and FN are the scores of true positive, true negative, false positive and false negative results, respectively. The higher the AUC, the more effective the classification.

Table S1: Global Diagnostic imaging Sites for Autism Spectrum Disorder from the ABIDE library were used in this study

Demographics of 496 Subjects

ABIDE-I

**Sites**	**Type**	**Count**	**Age**	**M/F**
**LEU**	ASD	27	12-32	25/02
	HC	31	12-32	28/03
**NYU**	ASD	72	7-39	65/07
	HC	100	6-31	78/22
**UCL**	ASD	43	10-16	40/03
	HC	41	9-13	38/03
**UOM**	ASD	41	8-18	37/04
	HC	63	8-28	55/08
**USM**	ASD	42	11-50	42/00
	HC	36	8-39	36/00
**Sites**	ASD Male	ASD Female	HC Male	HC Female
	209	16	235	36
05	225		271	
	**= 496**			

Demographics of 500 Subjects

ABIDE-II

**Sites**	**Type**	**Count**	**Age**	**M/F**
**BNI**	ASD	29	18-62	29/00
	HC	26	18-64	26/00
**GU**	ASD	43	8-13	28/15
	HC	40	8-13	30/10
**KKI1**	ASD	44	8-13	32/12
	HC	104	8-13	90/14
**NYU1**	ASD	45	5-34	40/05
	HC	29	5-23	27/02
**SDSU**	ASD	31	7-18	27/04
	HC	21	8-17	21/00
**OHSU**	ASD	34	7-15	26/08
	HC	54	8-14	31/23
**Sites**	ASD Male	ASD Female	HC Male	HC Female
	182	44	225	49
06	226		274	
	**= 500**			

Cumulative record of Datasets for ABIDE-I and ABIDE-II.

	**Sites**	**ASD Male**	**ASD Female**	**HC Male**	**HC Female**
**ABIDE-I**	05	209	16	235	36
**ABIDE-II**	06	182	44	225	49
		391	60	460	85
**Total**	11	**Total ASD = 451**		**Total HC = 545**	
		**= 996**			

ABIDE-I: LEU = University of Leuven; NYU = New York University Langone Medical Center; UCL = University California Log Angeles; UOM = University of Michigan; USM = University of Utah School of Medicine. **ABIDE-II:** BNI = Barrow Neurological Institute; GU = Georgetown University; KKI1 = Kennedy Krieger Institute; NYU1 = New York University Langone Medical Center; SDSU = San Diego State University; OHSU = Oregon Health & Science University

Table S2: Scanner Acquisition parameters and experimental settings of 11 sites from ABIDE-I & II

**Repository**	**Sites**	**MRI Vendor**	**TR [s]**	**TE [msec]**	**Vol**	**FA [°]**
**ABIDE-I**	**LEU**	Phillips	1.66	33	250	90
	**NYU**	Siemens	2	15	180	90
	**UCL**	Siemens	3	28	120	90
	**UOM**	SIGNA	2	30	300	90
	**USM**	Siemens	2	28	240	90
**ABIDE-II**	**BNI**	INGENIA	3	25	120	80
	**GU**	Siemens	2	30	154	90
	**KKI1**	Phillips	2.5	30	156	75
	**NYU1**	Siemens	2	15	180	90
	**SDSU**	MR750	2	30	180	90
	**OHSU**	Siemens	2.5	30	120	90

TR = Repetition Time; **TE** = Time to Echo; **Vol** = Volume; **FA** = Flip angle; ° = Degree; **s** = Seconds; **msec** = Milliseconds

Table S3.1. Individual site classification results of all the components of DMN network.

Sites	Components	Accuracy	AUC	Specificity	Sensitivity
**BNI**	07	60%	60.20%	58.33%	61.29%
	17	70.91%	67.64%	70.83%	70.97%
	19	63.64%	60.08%	63.64%	63.64%
	26	61.82%	40.72%	77.78%	58.70%
**GU**	07	65.06%	65.70%	62.79%	67.50%
	17	62.65%	55.81%	60.98%	64.29%
	19	62.65%	56.10%	60%	65.79%
	26	67.47%	65.12%	67.57%	67.39%
**KKI**	07	77.7%	83.48%	93.83%	58.21%
	17	74.32%	81.49%	92.31%	54.29%
	19	74.32%	80.35%	91.25%	54.41%
	26	80.41%	79.55%	87.88%	65.31%
**LEU**	07	68.97%	71.21%	74.07%	64.52%
	17	67.24%	70.85%	67.65%	66.67%
	19	63.79%	58.30%	66.67%	60.71%
	26	63.79%	54%	65.62%	61.54%
**NYU**	07	63.95%	58.83%	63.97%	63.89%
	17	62.21%	50.53%	62.96%	59.46%
	19	61.63%	62.85%	73.61%	53%
	26	61.63%	57.49%	66.67%	54.29%
**NYU1**	07	74.32%	73.79%	66.67%	79.55%
	17	67.57%	61.61%	64.71%	68.42%
	19	66.22%	62.22%	56.67%	72.73%
	26	75.68%	71.03%	76.19%	75.47%
**OHSU**	07	68.18%	54.47%	67.11%	75%
	17	71.59%	66.50%	71.01%	73.68%
	19	67.05%	42.37%	66.23%	72.73%
	26	67.05%	60.89%	71.93%	58.06%
**SDSU**	07	65.38%	50.84%	54.05%	93.33%
	17	71.15%	72.35%	66.67%	73.53%
	19	69.23%	58.06%	63.16%	72.73%
	26	67.31%	72.35%	57.14%	79.14%
**UCL**	07	67.86%	65.34%	81.82%	62.90%
	17	59.52%	55.53%	56.86%	63.64%
	19	64.29%	56.27%	70.37%	61.40%
	26	63.10%	62.34%	60.87%	65.79%
**UOM**	07	66.35%	52.65%	67.95%	61.54%
	17	65.38%	55.28%	68.49%	58.06%
	19	61.54%	58.92%	68.85%	51.16%
	26	63.46%	61.13%	72.73%	53.06%
**USM**	07	64.1%	57.08%	64.29%	64%
	17	71.79%	73.15%	70.59%	72.73%
	19	64.1%	61.64%	60%	68.42%

	26	64.1%	60.45%	61.76%	65.91%

Table S3.2. Individual site classification results of all the components of SMN network.

Sites	Components	Accuracy	AUC	Specificity	Sensitivity
**BNI**	01	67.27%	61.41%	66.67%	67.74%
	05	63.64%	57.69%	63.64%	63.64%
	06	67.27%	69.63%	64.29%	70.37%
	08	60%	58.62%	58.33%	61.29%
	10	65.45%	46.42%	81.82%	61.36%
	21	60%	64.99%	56.25%	65.22%
**GU**	01	65.06%	67.97%	61.70%	69.44%
	05	65.06%	58.08%	65.71%	64.58%
	06	61.45%	59.94%	60.53%	62.22%
	08	68.67%	62.73%	67.50%	69.77%
	10	62.65%	62.21%	60.47%	65%
	21	63.86%	63.84%	63.89%	63.83%
**KKI**	01	74.32%	78.58%	90.24%	54.55%
	05	78.38%	81.99%	91.86%	59.68%
	06	79.73%	82.98%	88.54%	63.46%
	08	67.57%	75.72%	91.18%	47.50%
	10	79.05%	81.56%	90.11%	61.40%
	21	70.27%	74.83%	92.86%	50%
**LEU**	01	68.97%	67.14%	78.26%	62.86%
	05	65.52%	61.17%	72%	60.61%
	06	60.34%	59.50%	61.76%	58.33%
	08	62.07%	54.84%	71.43%	56.76%
	10	67.24%	65.83%	68.75%	65.38%
	21	67.24%	64.99%	65%	72.22%
**NYU**	01	62.21%	56.06%	63.36%	58.54%
	05	63.95%	59.67%	69%	56.94%
	06	57.56%	57.89%	69.01%	49.50%
	08	61.63%	56.13%	65.74%	54.69%
	10	60.47%	61.69%	63.79%	53.57%
	21	66.28%	58.49%	66.15%	66.67%
**NYU1**	01	68.92%	68.20%	60%	75%
	05	67.57%	47.13%	64.71%	68.42%
	06	64.86%	45.52%	61.54%	65.57%
	08	70.27%	48.97%	88.89%	67.69%
	10	62.16%	65.36%	51.35%	72.97%
	21	62.16%	55.71%	51.28%	74.29%
**OHSU**	01	68.18%	58.61%	71.67%	60.71%
	05	64.77%	62.42%	73.47%	53.85%
	06	69.32%	55.01%	70.15%	66.67%
	08	62.5%	46.08%	64.38%	53.33%
	10	60.23%	59.26%	70.21%	48.78%
	21	62.5%	66.12%	74.42%	51.11%
	01	67.31%	62.67%	61.11%	70.59%

**SDSU**	05	65.38%	59.75%	56%	74%
	06	61.54%	64.06%	52%	70.37%
	08	67.31%	67.59%	70%	66.67%
	10	65.38%	55.30%	57.14%	70.97%
	21	69.23%	65.59%	61.90%	74.19%
**UCL**	01	61.9%	56.38%	60.98%	62.79%
	05	64.29%	56.89%	78.95%	60%
	06	66.67%	60.81%	93.33%	60.87%
	08	59.52%	40.73%	73.33%	56.52%
	10	63.10%	62.28%	63.89%	62.50%
	21	64.29%	65.68%	62.79%	65.85%
**UOM**	01	64.42%	52.73%	66.67%	57.69%
	05	71.15%	74.29%	85.11%	59.65%
	06	71.15%	66.86%	74.63%	64.86%
	08	62.5%	62.83%	70%	52.27%
	10	63.46%	64.85%	72.73%	53.06%
	21	61.54%	23.46%	62.64%	53.85%
**USM**	01	71.79%	73.21%	66.67%	77.78%
	05	64.1%	58%	60.53%	67.50%
	06	66.67%	61.57%	61.36%	73.53%
	08	73.08%	69.18%	70.27%	75.61%
	10	67.95%	67.39%	64.10%	71.79%
	21	67.95%	68.85%	64.86%	70.73%

Table S3.3. Individual site classification results of all the components of VSN network.

Sites	Components	Accuracy	AUC	Specificity	Sensitivity
**BNI**	11	67.27%	56.63%	66.67%	67.74%
	13	63.64%	65.65%	60%	68%
	15	67.27%	54.64%	90%	62.22%
	22	65.45%	55.04%	68.42%	63.89%
	24	72.73%	74.80%	82.35%	68.42%
	25	60%	58.22%	56.25%	65.22%
**GU**	11	64%	55.93%	62.50%	65.12%
	13	61.45%	60%	61.11%	61.70%
	15	63.86%	66.45%	63.16%	64.44%
	22	64%	60.64%	61.90%	65.85%
	24	65.06%	60%	63.41%	66.67%
	25	75.9%	74.36%	77.78%	74.47%
**KKI**	11	80.41%	83.17%	90.32%	63.64%
	13	71.62%	78.61%	88.75%	51.47%
	15	79.05%	84.24%	91.95%	60.66%
	22	77.02%	81.75%	93.75%	57.35%
	24	79.72%	84.20%	92.05%	61.67%
	25	75%	78.08%	89.41%	55.56%
**LEU**	11	63.79%	52.69%	85.71%	56.82%
	13	65.51%	62.37%	64.10%	68.42%
	15	63.79%	62.96%	69.23%	59.38%

	22	63.79%	61.41%	69.23%	59.38%
	24	62.06%	54.12%	68%	57.58%
	25	63.79%	62.84%	64.71%	62.50%
**NYU**	11	62.79%	49.62%	62.33%	65.38%
	13	66.86%	64.75%	67.48%	65.31%
	15	66.86%	62.54%	70.48%	61.19%
	22	62.79%	51.79%	62.86%	62.50%
	24	62.2%	46.10%	62.07%	62.96%
	25	65.11%	52.47%	65.87%	63.04%
**NYU1**	11	70.27%	76.09%	60%	79.49%
	13	64.86%	51.57%	58.82%	66.67%
	15	68.91%	55.94%	66.67%	69.64%
	22	67.56%	65.75%	58.06%	74.42%
	24	64.86%	53.95%	57.14%	67.92%
	25	74.32%	71.80%	67.86%	78.26%
**OHSU**	11	65.9%	35.40%	64.63%	83.33%
	13	64.77%	42.92%	65.75%	60%
	15	63.64%	54.08%	67.74%	53.85%
	22	62.5%	59.37%	69.81%	51.43%
	24	67.04%	57.46%	69.23%	60.87%
	25	60.22%	57.95%	69.39%	48.72%
**SDSU**	11	61.53%	60.06%	52.17%	68.97%
	13	65.38%	65.44%	56.52%	72.41%
	15	69.23%	63.59%	60.87%	75.86%
	22	67.3%	53.61%	62.50%	69.44%
	24	73.07%	76.65%	62.96%	84%
	25	63.46%	63.59%	53.12%	80%
**UCL**	11	64.28%	67.39%	63.41%	65.12%
	13	70.23%	69.43%	70%	70.45%
	15	59.52%	33.41%	55.07%	80%
	22	69.04%	66.19%	65.96%	72.97%
	24	72.61%	74.82%	68%	79.41%
	25	73.8%	71.81%	85.19%	68.42%
**UOM**	11	65.38%	63.22%	76.47%	54.72%
	13	68.26%	74.72%	82.61%	56.90%
	15	58.65%	44.06%	62.20%	45.45%
	22	65.38%	63.72%	75.47%	54.90%
	24	65.38%	52.46%	67.53%	59.26%
	25	61.53%	53.58%	63.86%	52.38%
**USM**	**11**	62.82%	58%	58.14%	68.57%
	**13**	62.82%	60.71%	61.29%	63.83%
	**15**	66.67%	62.43%	61.90%	72.22%
	**22**	60.25%	61.24%	57.58%	62.22%
	**24**	70.51%	63.62%	70.97%	70.21%
	**25**	65.38%	63.82%	60.98%	70.27%

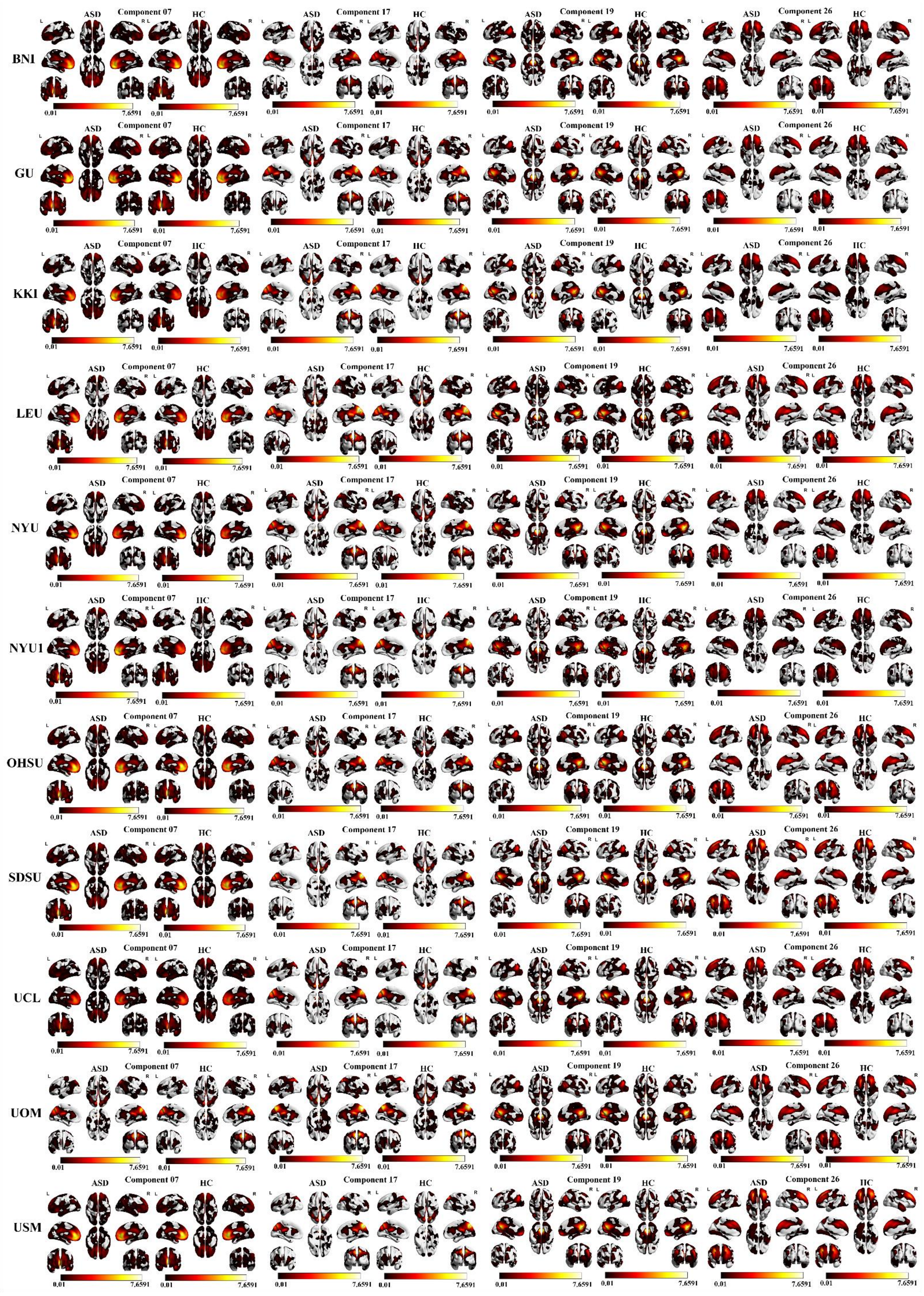

Figure S1. DMN components (07, 17, 19, 26) across 11 independent sites. Grouped as posterior vs anterior, selected based on its superior classification performance. ASD and HC activation maps are visualized as axial slices with color intensity reflecting the strength of activation.

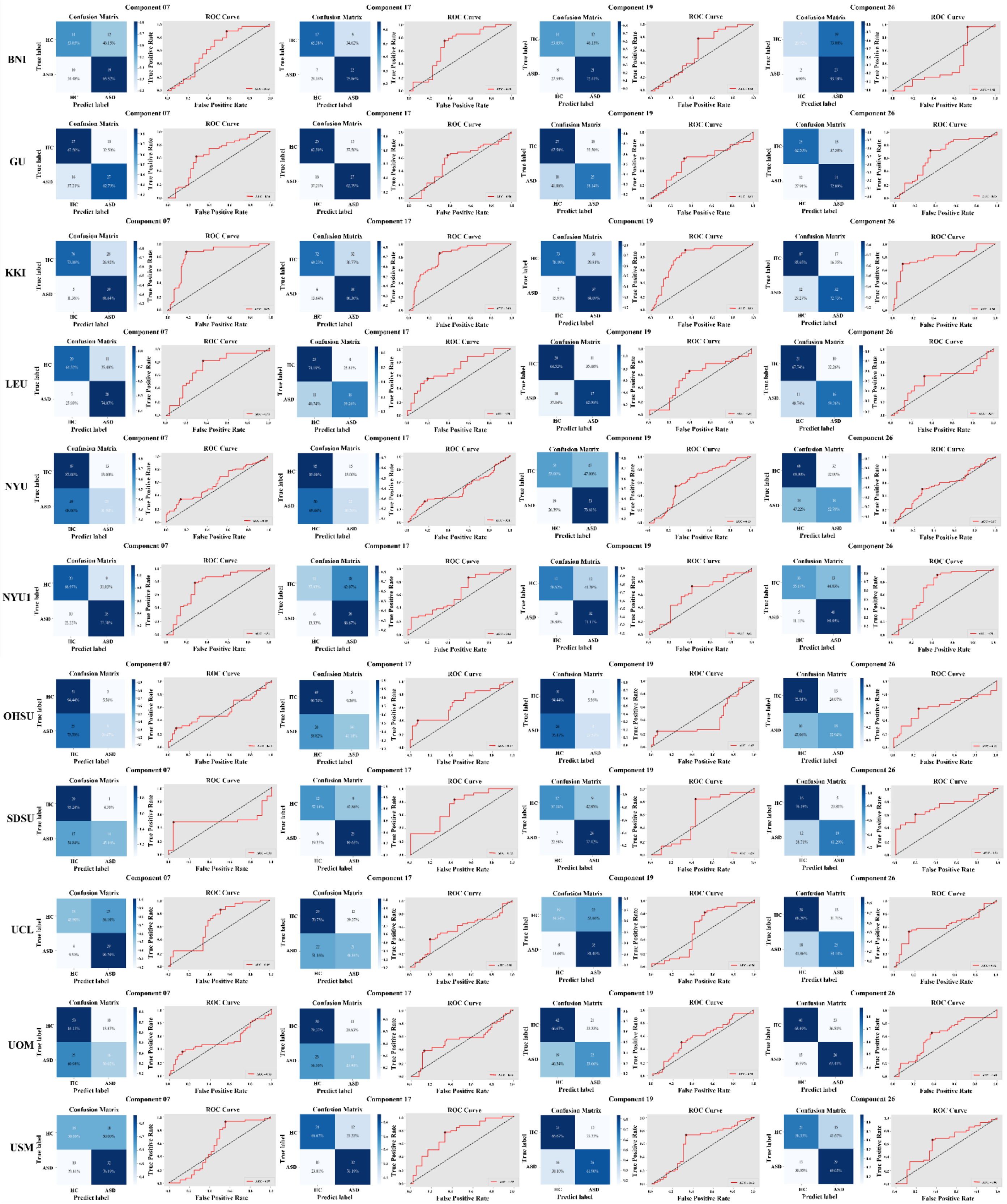

Figure S2. Classification performance of DMN ICA components (07, 17, 19, 26) across 11 independent sites. Classification performance of components includes a confusion matrix and ROC curve between ASD and healthy controls.

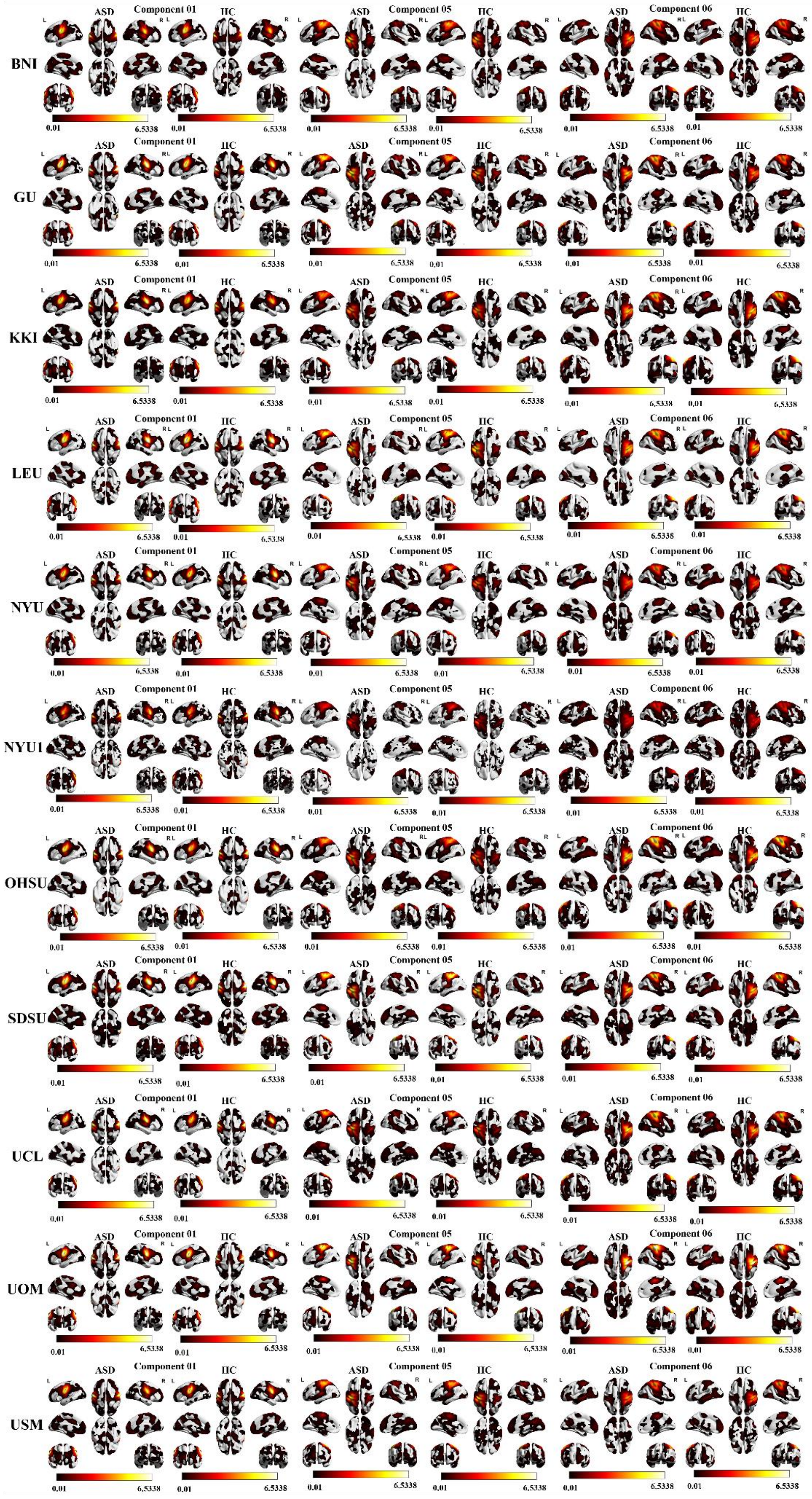

Figure S3. Visualization of SMN components (01, 05, 06) across 11 independent sites. Activation patterns involve regions within the postcentral gyrus, superior frontal gyrus and paracentral lobule. Spatial maps are shown for ASD and HC groups, with each row representing one site and axial slices visualizing activation intensity.

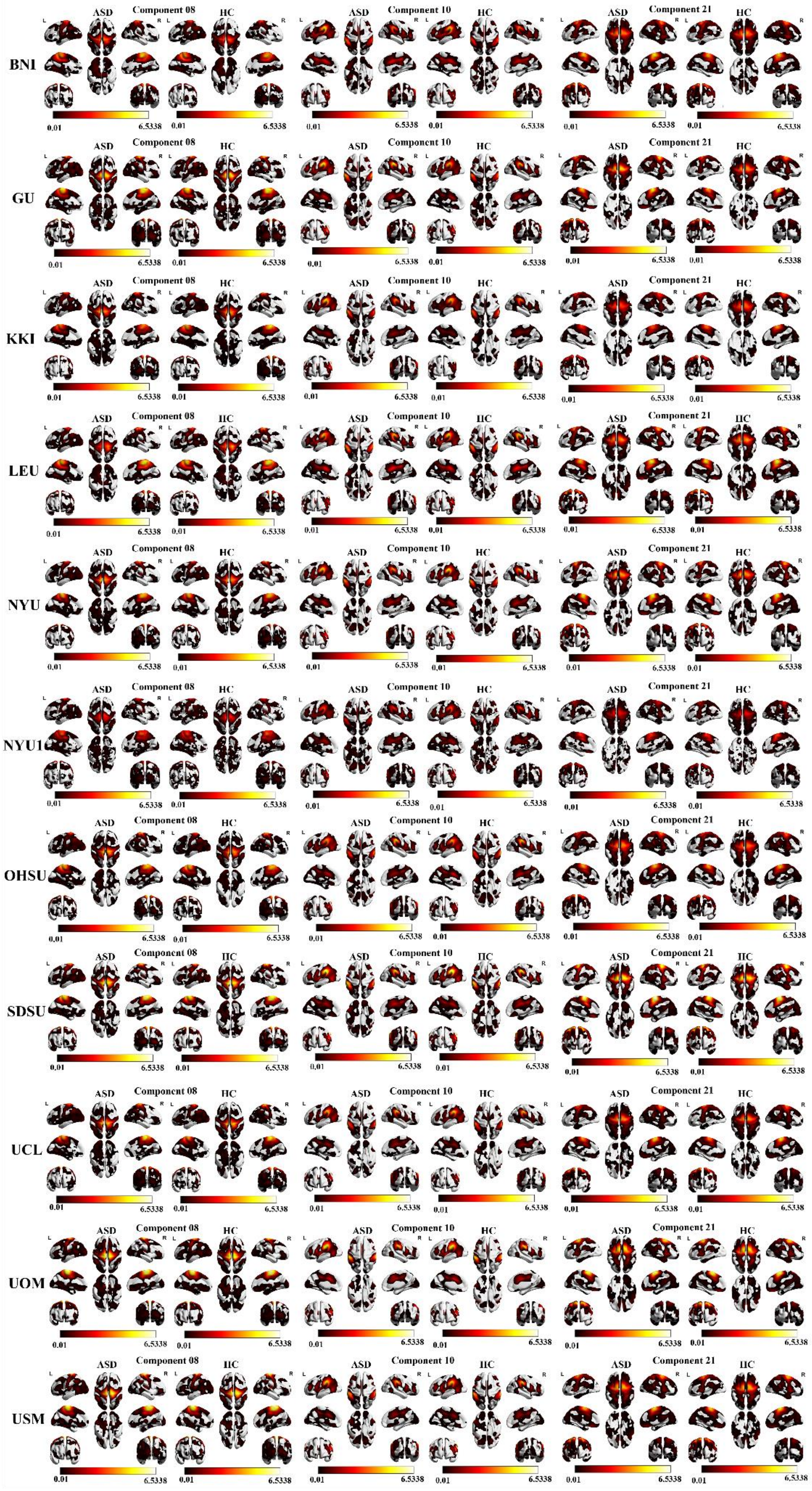

Figure S4. Visualization of SMN components (08, 10, 21) across 11 independent sites. Activation patterns involve regions within the postcentral gyrus, superior frontal gyrus and paracentral lobule. Spatial maps are shown for ASD and HC groups, with each row representing one site and axial slices visualizing activation intensity.

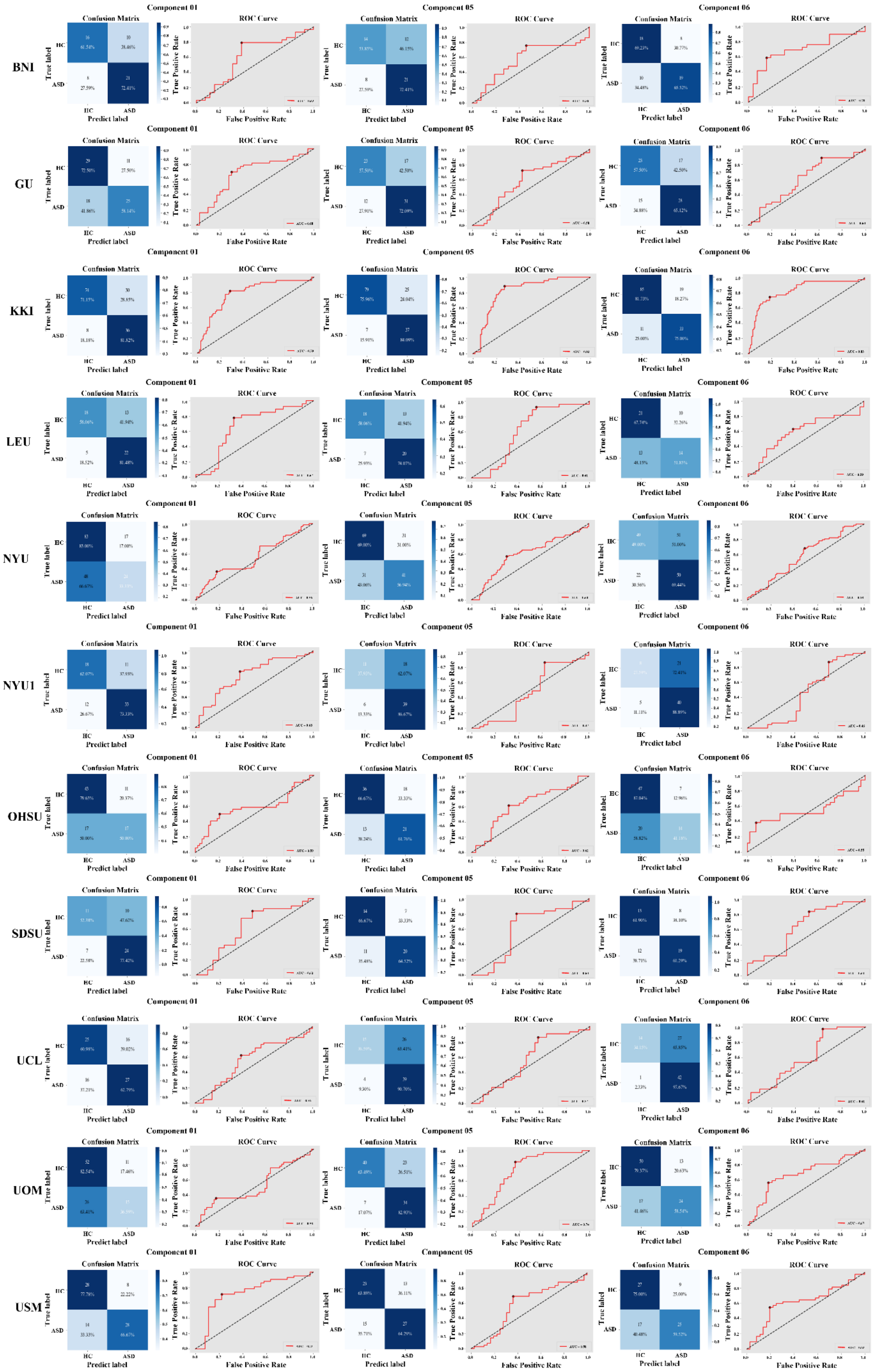

Figure S5. Classification performance of SMN ICA components (01, 05, 06) across 11 independent sites. Classification performance of components includes a confusion matrix and ROC curve between ASD and healthy controls.

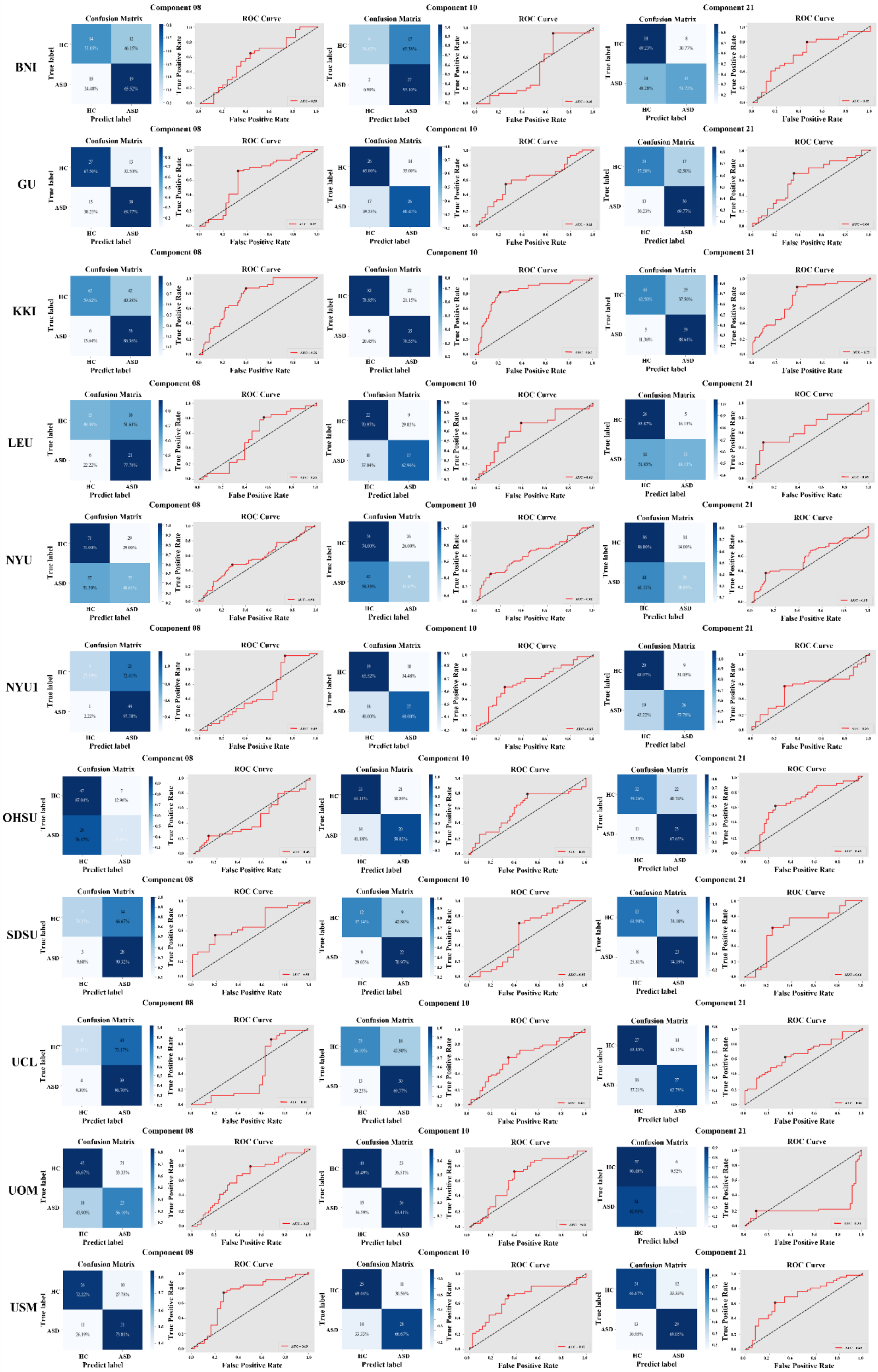

Figure S6. Classification performance of SMN ICA components (08, 10, 21) across 11 independent sites. Classification performance of components includes a confusion matrix and ROC curve between ASD and healthy controls.

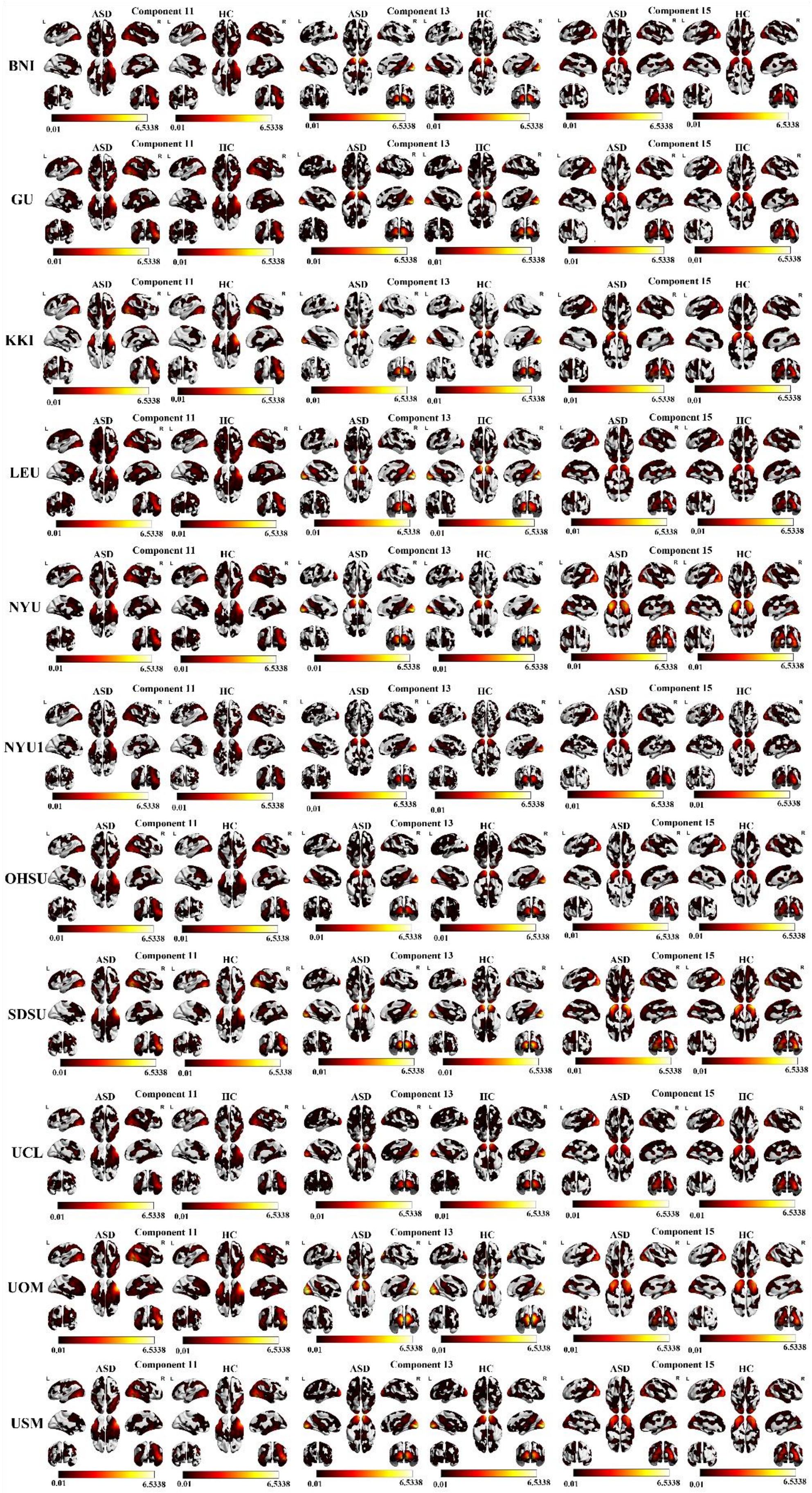

Figure S7. Visualization of VSN components (11, 13, 15) across 11 independent sites. Showing site-specific activation patterns in the occipital lobe, fusiform gyrus, temporal lobe, cerebellar posterior lobe of VSN components. Each row displays ASD and HC axial activation maps, with color intensity indicating activation strength.

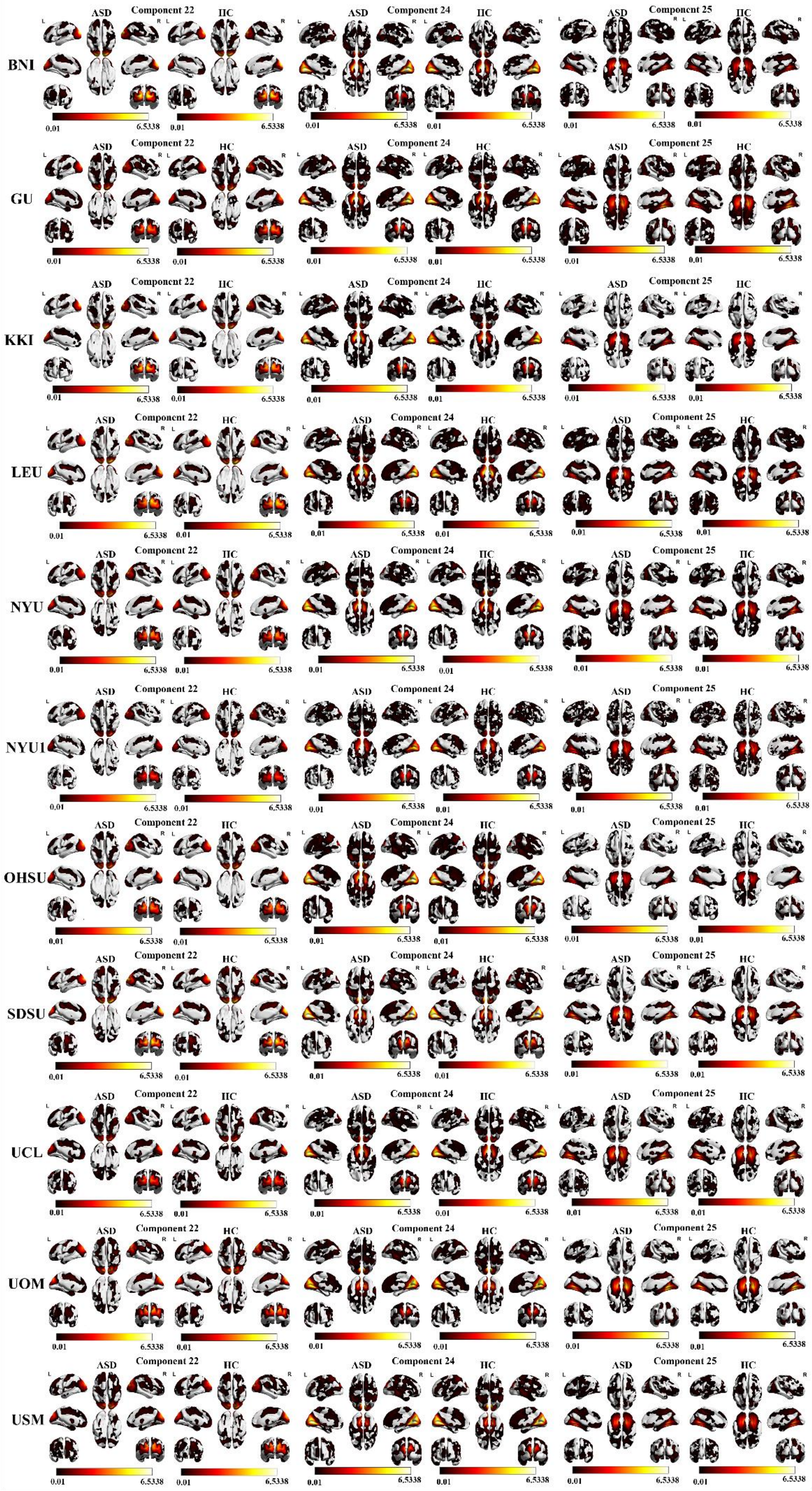

Figure S8. Visualization of VSN components (22, 24, 25) across 11 independent sites. Showing site-specific activation patterns in the occipital lobe, fusiform gyrus, temporal lobe, cerebellar posterior lobe of VSN components. Each row displays ASD and HC axial activation maps, with color intensity indicating activation strength.

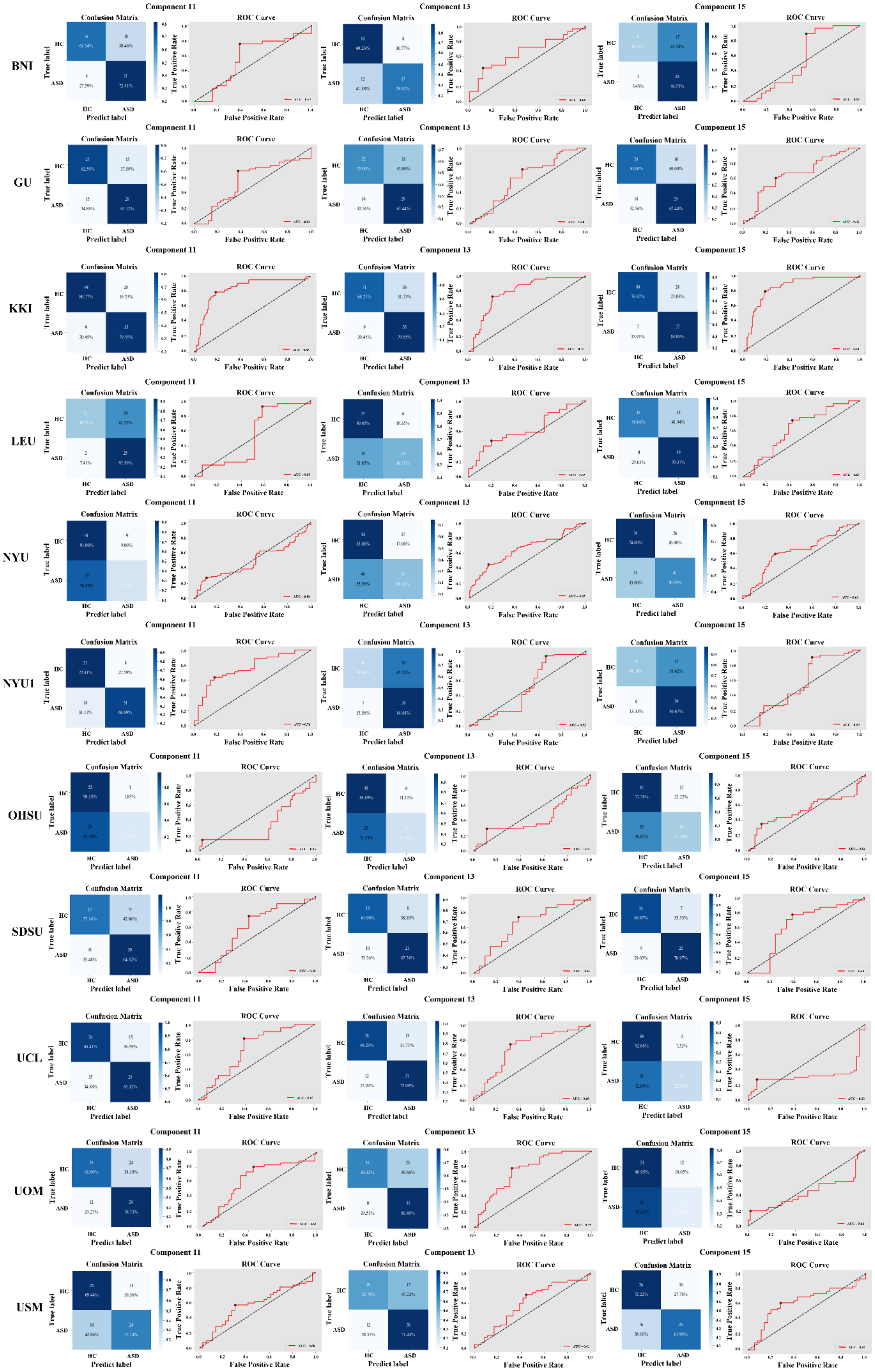

Figure S9. Classification performance of VSN ICA components (11, 13, 15) across 11 independent sites. Classification performance of components includes a confusion matrix and ROC curve between ASD and healthy controls.

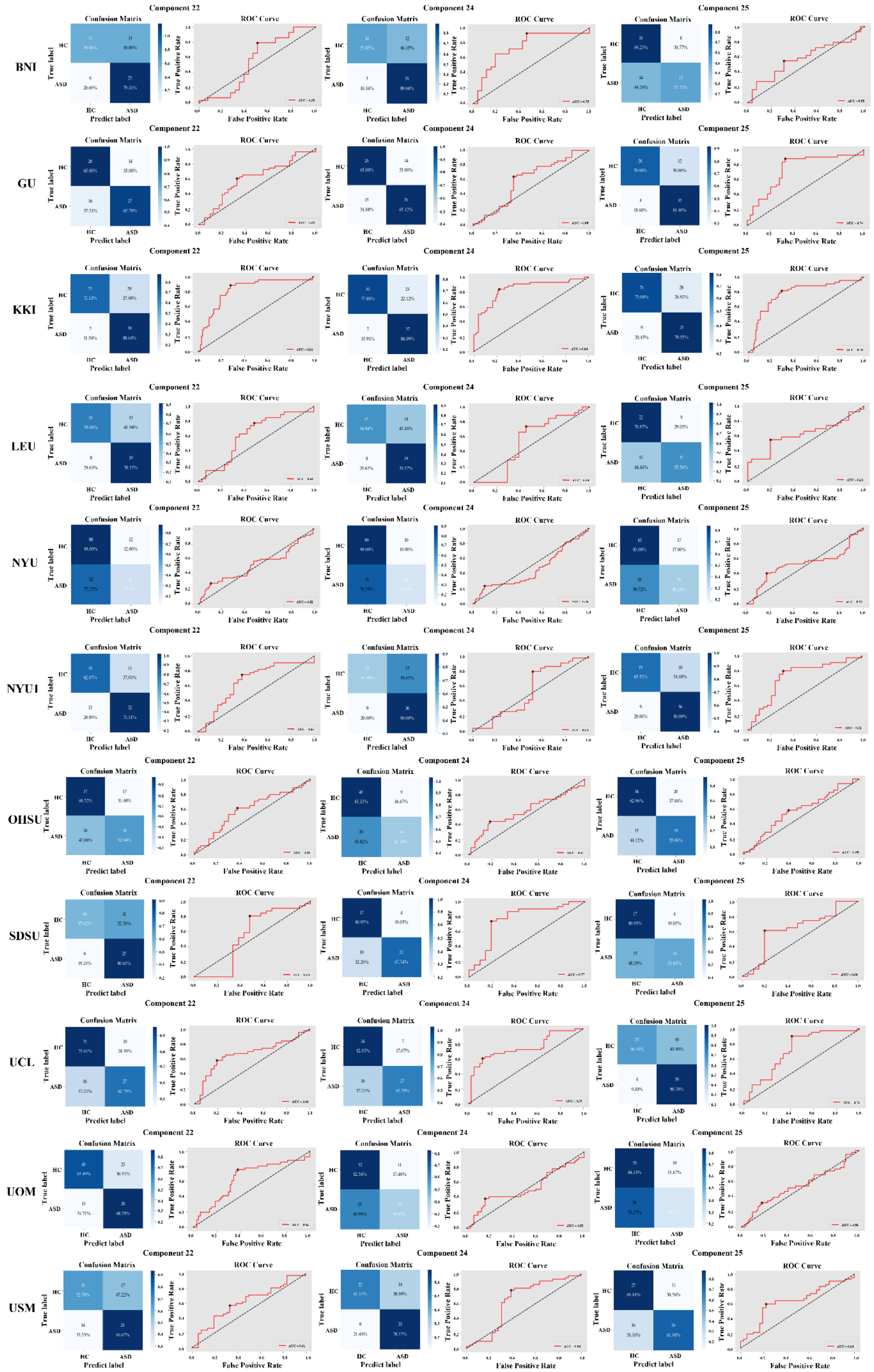

Figure S10. Classification performance of VSN ICA components (22, 24, 25) across 11 independent sites. Classification performance of components includes a confusion matrix and ROC curve between ASD and healthy controls.

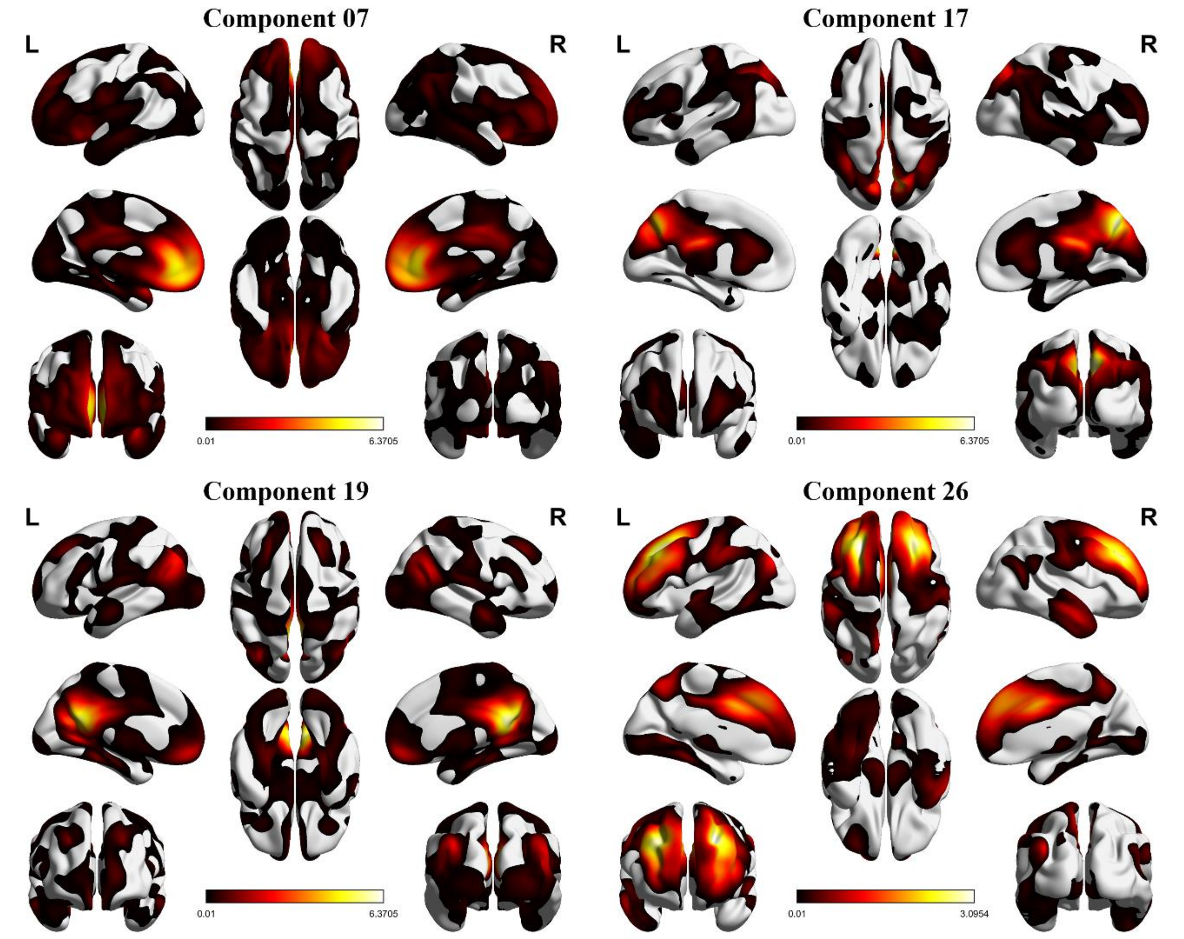

Figure S11. Visualization of the average spatial brain maps of DMN network components (07, 17, 19, 26). Components are visualized as axial slices displaying spatial patterns of brain activity, with color intensity reflecting the strength of activation.

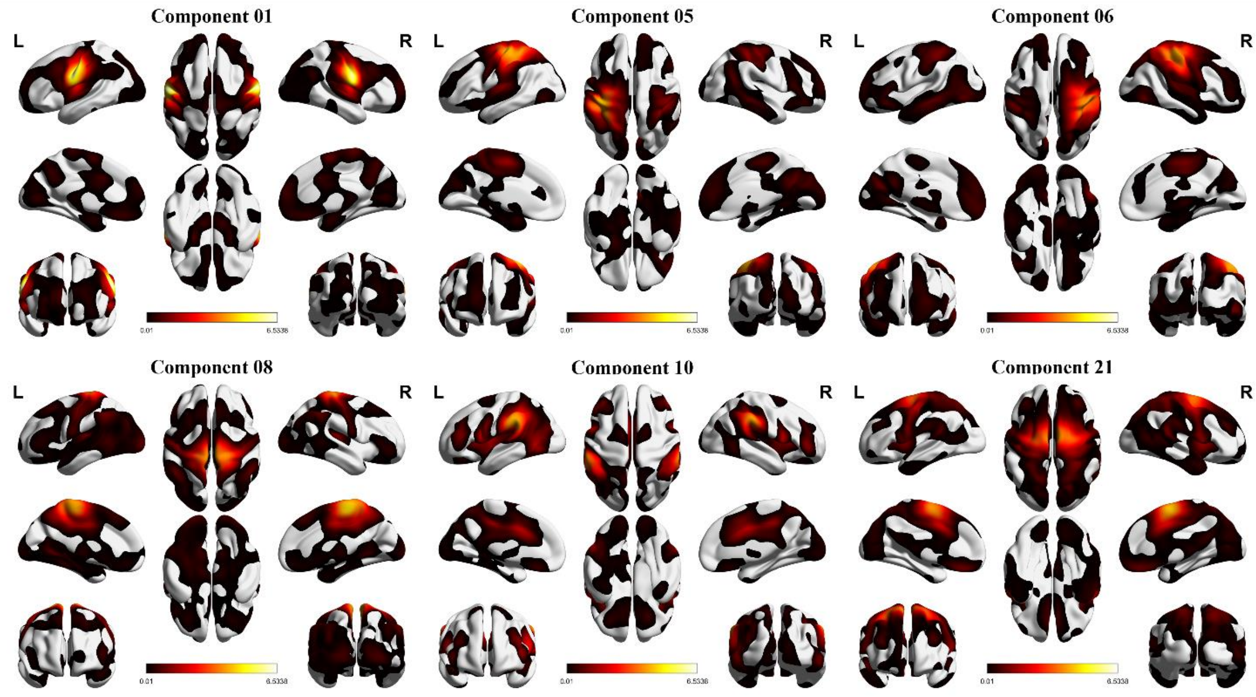

Figure S12. Visualization of the average spatial brain maps of SMN network components (01, 05, 06, 08, 10, 21). Components are visualized as axial slices displaying spatial patterns of brain activity, with color intensity reflecting the strength of activation.

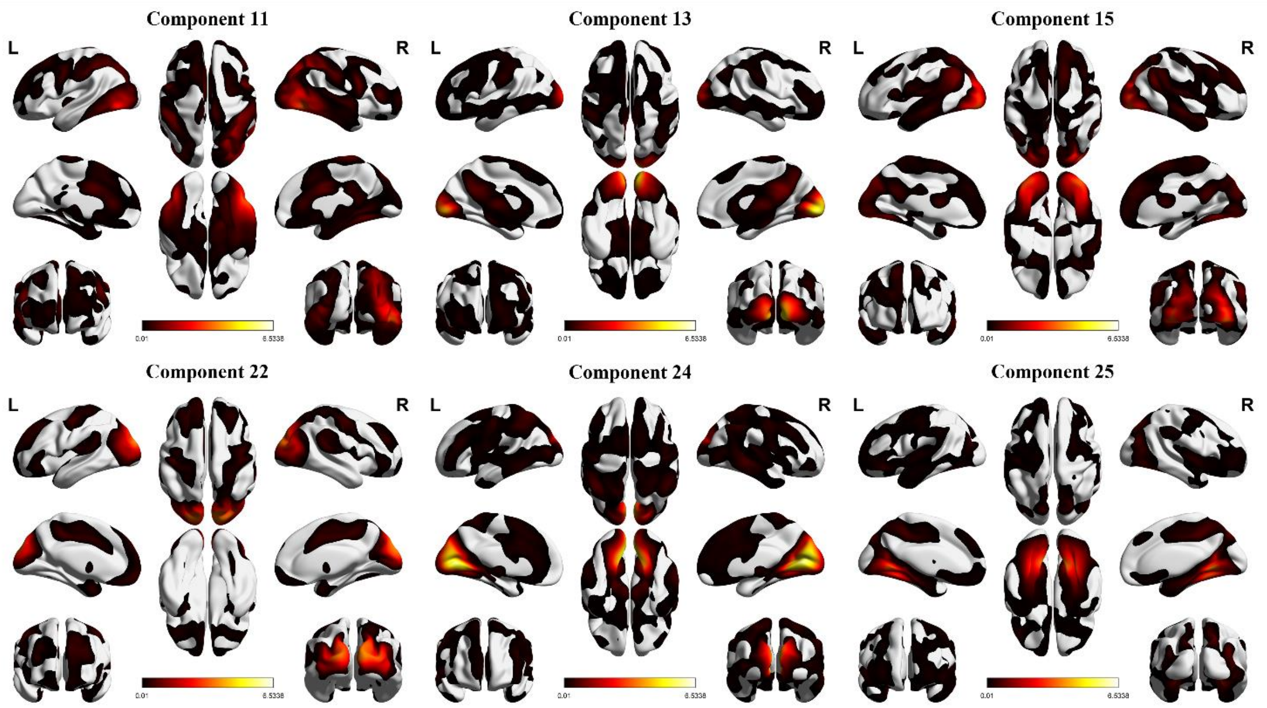

Figure S13. Visualization of the average spatial brain maps of VSN network components (11, 13, 15, 22, 24, 25). Components are visualized as axial slices displaying spatial patterns of brain activity, with color intensity reflecting the strength of activation.
